# Leonurine Ameliorates Doxorubicin‐Induced Cardiotoxicity via STING/NF‐κB/NLRP3 Inflammasome Signaling Pathway

**DOI:** 10.1002/advs.75912

**Published:** 2026-06-12

**Authors:** Wang Jun, Chen Xiaoyang, Xu Jianglin, Yang Zhi, Tao Meijiao, He Jiale, Zhao Jiangfeng, Hei Xuanding, Xu Xuegong, Wang Wei, Li Chun

**Affiliations:** ^1^ School of Basic Medicine Guangzhou University of Chinese Medicine Guangzhou China; ^2^ State Key Laboratory of Traditional Chinese Medicine Syndrome Guangzhou University of Chinese Medicine Guangzhou China; ^3^ Institute of Formula and Syndrome School of Pharmaceutical Sciences Guangzhou University of Chinese Medicine Guangzhou China; ^4^ Chinese Medicine Guangdong Laboratory Hengqin China; ^5^ Science and Technology Innovation Center Zhengzhou Hospital of Traditional Chinese Medicine (Zhengzhou Hospital of TCM Affiliated to Henan University of Chinese Medicine) Zhengzhou China; ^6^ Henan Provincial Key Laboratory of Traditional Chinese Medicine for Cardiovascular Diseases Zhengzhou Hospital of Traditional Chinese Medicine Zhengzhou China; ^7^ Department of Cardiovascular Medicine Zhengzhou Traditional Chinese Medicine Hospital Zhengzhou China; ^8^ Department of Cardiology The Second Affiliated Hospital of Guangzhou University of Chinese Medicine Guangzhou China; ^9^ Institute of Traditional Chinese Medicine Beijing University of Chinese Medicine Beijing China

**Keywords:** doxorubicin‐induced cardiomyopathy, leonurine, NLRP3, sting, vesicle

## Abstract

Doxorubicin‐induced cardiomyopathy (DIC) remains a dose‐limiting clinical challenge. This study reveals that cardiac vascular endothelial cells (CVECs) act as initial sensors of doxorubicin cardiotoxicity: circulating doxorubicin activates the cGAS‑STING pathway in CVECs, triggering NLRP3 inflammasome‑mediated pyroptosis and release of pathogenic extracellular vesicles that induce mitochondrial dysfunction in neighboring cardiomyocytes, establishing a self‑perpetuating injury loop. Leonurine (LEO), a natural alkaloid, is identified as a direct STING inhibitor that specifically binds the TYR261 residue, blocking both STING oligomerization and STING‑TBK1 heterodimer formation—a mechanism distinct from known STING inhibitors. LEO exerts hierarchical dual protection: directly preserving cardiomyocyte mitochondria while primarily inhibiting endothelial STING to disrupt the pathogenic loop. This endothelial‑centric strategy shifts the therapeutic paradigm from direct cardiomyocyte protection to upstream endothelial intervention, establishing LEO as a promising candidate for DIC.

## 1 Introduction

Doxorubicin (DOX) remains a cornerstone of cancer therapy, yet its clinical utility is severely curtailed by dose‐dependent cardiotoxicity that progresses to irreversible cardiomyopathy and heart failure—a condition for which no effective targeted therapy exists [1–4]. Despite decades of research focused on mitigating direct cardiomyocyte injury, the fundamental question of how an initial myocardial insult escalates into progressive cardiac dysfunction remains unanswered [5–10]. This critical knowledge gap underscores the urgent need to redefine the cellular hierarchy and molecular circuitry driving Doxorubicin‐induced cardiomyopathy (DIC).

Emerging evidence implicates innate immune signaling in DIC pathogenesis, yet the prevailing cardiomyocyte‐centric view has largely overlooked the potential role of cardiac vascular endothelial cells (CVECs) as active disease instigators [11–15]. CVECs constitute the first cellular barrier encountered by circulating DOX and are essential for myocardial homeostasis, yet their contribution to DIC initiation and propagation remains unexplored [16–20]. Specifically, whether CVECs function as initial sensors of DOX injury and whether they orchestrate a pathogenic dialogue with cardiomyocytes are pivotal unanswered questions [21–24].

This study uncovers that CVECs serve as the primary sensors and amplifiers of DOX cardiotoxicity. Circulating DOX directly activates the cGAS (Cyclic GMP‐AMP synthase)/STING (Stimulator of interferon genes) pathway in CVECs, triggering NLRP3 inflammasome‐mediated pyroptosis and the release of pathogenic extracellular vesicles that propagate mitochondrial damage to neighboring cardiomyocytes—establishing a previously unrecognized self‐perpetuating injury loop [18–20]. This endothelial‐centric mechanism fundamentally revises the traditional paradigm of DIC pathophysiology.

Furthermore, Leonurine (LEO), a natural alkaloid from *Leonurus japonicus*, is identified as a direct STING inhibitor with a novel dual mechanism [25, 26]. Through comprehensive structural and biochemical analyses, LEO is demonstrated to specifically bind the previously unexplored TYR261 residue of STING, simultaneously blocking STING oligomerization and STING‐TBK1 heterodimer formation. This non‐covalent binding mode is distinct from known STING inhibitors and offers unique therapeutic advantages. By disrupting the endothelial pathogenic loop at its initiation, LEO effectively preserves cardiac function, establishing a new paradigm for DIC treatment centered on endothelial protection.

## 2 Materials and Methods

### 2.1 Chemicals and Reagents

Primary antibodies for cGAS, STING, P‐STING, TBK1, P‐TBK1, NF‐κB, P‐NF‐κB, NLRP3, GSDMD‐FL, GSDMD‐NT, CD68, F4/80, COL‐I, α‐SMA, β‐ACTIN, and GAPDH were obtained from Cell Signaling Technology (Denvers, MA, USA). Antibody for dsDNA (AC‐30‐10) was obtained from Progen (Germany, Heidelberg). The Reactive oxygen (ROS) Fluorometric Assay Kit (E‐BC‐F005) and the Annexin V‐FITC/PI Apoptosis Kit (E‐CK‐A211) were obtained from Elabscience Biotechnology Co., Ltd. (Wuhan, China). SiRNA‐STING and SiRNA‐Con were obtained from Kidan Bio Co. Ltd, Guangzhou, China. The plasmids pcADV‐EF1‐mNeonGreen‐CMV‐STING and pcADV‐EF1‐mNeoGreen‐CMV‐MCS (control) were obtained from Heyuan Biotechnology Co., Ltd, Shanghai, China. Leonurine (CAS: 24697‐74‐3) was obtained from Ruifen Si Biotechnology Co., Ltd, Chengdu, China. Doxorubicin hydrochloride (25316‐40‐9), Dexrazoxane (DEX, 149003‐01‐0), Disulfiram (DSF, B70221), C‐176 (314054‐00‐7), 2′,3′‐cGAMP sodium salt (2734858‐36‐5), and Pravastatin sodium (PRA, 81093‐37‐0) were sourced from Yuanye Biotechnology Co., Ltd, Shanghai, China. Wheat Germ Agglutinin (WGA, 13310), Hematoxylin‐Eosin (HE, g1120), MASSON (g1340), and TdT‐mediated dUTP nick end labeling (Tunel, T2195) kits were procured from Solabio Technology Co., Ltd, Beijing, China. The following kits were procured from Elabscience Biotechnology Co., Ltd.: Creatine kinase (CKMB, E‐EL‐M0355), Lactate dehydrogenase (LDH, E‐BC‐K046‐M), Myoglobin (Myo, E‐EL‐R0053), Troponin T (cTNT, E‐EL‐M1801), Brain natriuretic peptide (BNP, E‐EL‐M0204), NT‐proBNP (E‐EL‐M0834), Cysteine protease 1 (CASP1, E‐EL‐M0201), Intercellular adhesion molecule 1 (ICAM‐1, E‐EL‐M3037), ICAM‐2 (E‐MSEL‐M0020), Vascular cell adhesion factor (VCAM‐1, E‐EL‐M1233), von willebrand factor (vWF, E‐EL‐M1247), Interleukin 18 (IL18, E‐EL‐M0730c), IL1β (E‐EL‐M0037c), Tumor Necrosis Factor (TNF‐α, E‐EL‐M3063), IL10 (E‐EL‐M0046), Extracellular acidification rate (ECAR, E‐BC‐F069), Oxygen consumption rate (OCR, E‐BC‐F068), Adenosine triphosphate (ATP) / Adenosine diphosphate (ADP) (E‐BC‐F004), Nicotinamide adenine dinucleotide (NAD+)/NADH (E‐BC‐K804‐M), Mitochondrial respiratory chain complex I‐V (E‐BC‐K149‐M, E‐BC‐K150‐M, E‐BC‐K836‐M, E‐BC‐K837‐M, E‐BC‐K838‐M), Aspartate aminotransferase (AST, E‐BC‐K236‐M), Alanine aminotransferase (ALT, E‐BC‐K235‐M), Total bilirubin (TBIL, E‐BC‐K760‐M), Albumin (ALB, E‐EL‐M0792), Creatinine (Cr, E‐BC‐K188‐M), Urea nitrogen (BUN, E‐BC‐K183‐M), CKMB (E‐EL‐M0355), cTNT (E‐EL‐M1801) and LDH (E‐BC‐K771‐M). Human STING protein (24013068P‐1) was obtained from Pujian Biotechnology Co., Ltd, Wuhan, China. Tyramide Signal Amplification (TSA) Kits (K1050, K1051, K1052) were obtained from APExBIO, Houston, USA. The Mouse Cardiac Microvascular Endothelial Cell (CMVEC) Isolation and Culture Kit (P‐CA‐714) were obtained from Pricella Biotechnology Co., Ltd, Wuhan, China. Streptavidin magnetic beads (YJ011) were obtained from Yamay Biomedical Technology Co., Ltd, Shanghai, China. Primers for mouse/human‐specific genes, including cGAS, STING, TBK1, NF‐κB, NLRP3, CASP1, GSDMD, IL18, IL1β, ANP, BNP, β‐MHC, MT‐CO1, MT‐CO2, and MT‐ATP, were sourced from Shenggong Bioengineering Co., Ltd (Shanghai, China) (Table S1). All other commercially available chemicals were of the highest quality.

### 2.2 Animals

Wild‐type (WT) mice (C57BL/6 background) were purchased from Charles River (Beijing, China). STING Knockout (KO) mice (C57BL/6 background) were kindly provided by Professor Changhui Liu from the School of Chinese Medicine, Guangzhou University of Chinese Medicine. All mice were housed in a barrier facility with a 12/12 h light/dark cycle, with free access to water and a standard chow diet. The animals were divided into the following groups: CON group, DOX group, LEO‐L group (low dose of LEO, 5 mg/kg/day), LEO‐M group (medium dose of LEO, 10 mg/kg/day), LEO‐H group (high dose of LEO, 20 mg/kg/day), PRA group (Pravastatin, 40 mg/kg/day), or C‐176 group (750 nM), and were treated for a total of 4 weeks. Except for the CON group, all other groups received a tail vein injection of DOX (20 mg/kg) according to the previously established method to induce a DIC model. After the aforementioned pretreatment, the mice were euthanized. Serum samples were collected and stored at −20°C for further analyses. Heart tissues were harvested and either stored at −80°C for biochemical and molecular biology analyses or fixed in 4% formalin for histological assessment and immunofluorescence detection. All animal care and experimental protocols adhered to and were approved by the guidelines of the Animal Ethics Committee of Guangzhou University of Chinese Medicine (approval No. 20240926009).

### 2.3 Cell Culture and Treatment

CMVEC were isolated according to the kit instructions, and their purity was verified through CD31 staining before subsequent passage culture and experiments. Human Umbilical Vein Endothelial Cells (HUVEC, CP‐H082) and H9C2 (CL‐0089) cell lines were obtained from Pricella Biotechnology Co., Ltd, Wuhan, China. To generate STING‐overexpressing cell lines, CMV‐STING‐OE (or CMV‐CON as a control) was introduced following the kit instructions. Similarly, STING knockdown cell lines were established by transfecting Si‐RNA (or a non‐targeting sequence as a control). Cells were cultured in DMEM with 10% FBS, 100 U/mL penicillin, and 0.1 mg/mL streptomycin at 37°C in a 5% CO2 humidified incubator. Culture reagents were sourced from Gibco, Carlsbad, CA, USA. Cells were exposed to serum‐free DMEM with either 300 nM DOX or 5 µM 2′,3′‐cGAMP for 24 h to establish a cell injury model. Cells were treated with varying concentrations of LEO (2.5, 5, 10 µM), PRA (0.5 µM), C‐176 (0.75 µM), or DSF (120 µM).

### 2.4 Methods for Extracellular Vesicles (EVs) Extraction and Identification

Extract EVs from endothelial cell culture media supernatant using ultracentrifugation. Initially, conduct sequential low‐speed centrifugation at 300×g, 2000×g, and 10 000×g to eliminate large particles, including cell debris and apoptotic bodies. Perform ultracentrifugation at speeds of 100 000×g or greater at 4°C for 70–120 min to isolate EVs. After resuspending the pellet in PBS, perform another round of ultracentrifugation for purification, ultimately yielding high‐purity EVs. This method is applicable to samples such as cell culture supernatants and serum, but strict control of temperature (4°C) and sterile operation is required to prevent degradation. Transmission electron microscopy (TEM) is considered the gold standard for morphological detection of EVs. The cup‐shaped or double‐layered membrane structure of EVs can be observed through uranyl acetate negative staining or cryo‐TEM techniques. Among these, cryo‐TEM avoids deformation caused by chemical fixation and directly presents EVs morphology in a near‐native state with a resolution of up to 1 nm. However, ample preparation is cumbersome and requires specialized equipment. Nanoparticle tracking analysis (NTA), on the other hand, tracks the real‐time motion trajectories of individual particles through laser scattering.

### 2.5 Western Blotting (WB)

Proteins were isolated from tissues or cells using RIPA buffer (Beyotime, Hangzhou) supplemented with protease inhibitors and quantified using a BCA assay (Dingguo Biology, Guangzhou).Samples at equal concentrations were combined with LDS buffer, separated using 8–15% SDS‐PAGE, and transferred onto PVDF membranes. Membranes were incubated in 5% non‐fat milk in TBS‐T at room temperature for 1.5 h to block them. Membranes were incubated overnight at 4°C with primary antibodies, followed by a 2‐hour incubation at room temperature with HRP‐conjugated secondary antibodies. Proteins were ultimately visualized using ECL kits (Millipore, Billerica, USA).Protein band intensities were analyzed with Image J software and normalized to β‐ACTIN or GAPDH.

### 2.6 Co‐Immunoprecipitation (CO‐IP) Assays

Cell or tissue samples were collected and lysed on ice for 30 min using a mild lysis buffer containing protease inhibitors, followed by centrifugation to obtain the supernatant. Biotin‐labeled bait protein antibodies were then added to the lysate and incubated overnight at 4°C to form antigen‐antibody complexes. Subsequently, streptavidin‐conjugated magnetic beads were added to capture the complexes via the high‐affinity streptavidin‐biotin interaction, with rotational incubation at 4°C for 1–2 h. The magnetic bead–complex conjugates were separated using a magnetic stand and washed with a low‐salt buffer to remove non‐specifically bound proteins. Finally, the target protein was eluted with an SDS‐containing elution buffer, separated by SDS‐PAGE, and its interacting partners were identified by Western blot. This method combines the efficient capture properties of the streptavidin‐biotin system, significantly enhancing the specificity and sensitivity of protein‐protein interaction studies.

### 2.7 Pull‐Down Assays

First, LEO was biotinylated using a commercial biotinylation reagent in accordance with the manufacturer's instructions. Subsequently, the biotinylated small molecule was incubated with samples such as cell lysates to allow potential binding proteins to form complexes with the small molecule. Thereafter, magnetic beads were used to capture the complexes, followed by multiple washes to remove non‐specifically bound proteins. Finally, the bound proteins were eluted from the magnetic beads using an elution buffer, and the proteins in the eluted fraction were identified and analyzed via Western Blot to determine the proteins that bind to the small molecule.

### 2.8 Real‐Time Quantitative Polymerase Chain Reaction (RT‐qPCR)

Use an RNA extraction kit for isolation. RNA undergoes reverse transcription to form complementary DNA (cDNA). The qPCR reaction system involves combining cDNA, target RNA sequence primers, and SYBR Green for PCR product detection. Refer to Table S1 for primer sequences. A thermal cycling PCR machine is necessary for conducting multiple qPCR cycles, and fluorescence data should be evaluated using the 2^−ΔΔCT^ method to determine target RNA expression levels.

### 2.9 Enzyme‐Linked Immunosorbent Assay (ELISA) Assay

Cytokines or metabolites concentrations, including IL18, IL1β, TNF‐α, IL10, and CASP1, were quantified in serum, tissue supernatants, and cell culture media via ELISA. The procedure was meticulously followed as per the manufacturer's guidelines.

### 2.10 CCK8 Assay

HUVECs or CMVEC were seeded into 96‐well plates at suitable densities and incubated at 37°C with 5% CO_2_ for 24 h to facilitate attachment. Various concentrations of LEO were incorporated into the culture medium, including a control group. Cells were cultured for 3, 6, 12, or 24 h.CCK‐8 reagent, constituting 10% of the total volume, was added to each well and incubated for 1–4 h to ensure full reaction. The optical density at 450 nm was measured using a microplate reader. Cell viability and proliferation were assessed by observing variations in optical density (OD) values.

### 2.11 Pathological Staining

Mouse heart tissue sections or cell slides were fixed in tissue fixation solution and washed three times with PBS. HE, Masson, and WGA staining were performed according to the manufacturers' protocols. Heart tissues were washed with normal saline, dehydrated, embedded in paraffin, and fixed in 10% buffered formalin. The samples were sectioned into 5 µm slices, deparaffinized, and stained with HE, Masson, and WGA for histological analysis. Visualization was performed using a panoramic scanning system (Olympus VS200, Tokyo, Japan) or a stereoscope (Olympus X70, Tokyo, Japan).

### 2.12 Immunohistochemistry (IHC) and Immunofluorescence (IF) Staining

Samples were fixed with paraformaldehyde and underwent microwave antigen retrieval. After PBS washes, permeabilization (1% Triton X‐100, 15 min) and blocking (5% BSA, 1 h) were performed. Endogenous peroxidase was quenched with 3% H_2_O_2_. Primary antibodies (1:200) and HRP‐conjugated secondary antibodies (1:200) were applied, followed by chromogenic/TSA (Tyramide Signal Amplification) staining. Nuclei were counterstained with DAPI/hematoxylin. Imaging and analysis were conducted using an Olympus fluorescence microscope or panoramic scanner. For fluorescence quantification, mean fluorescence intensity was measured using ImageJ software, with DAPI staining used to define nuclear regions and normalize target protein expression levels. For colocalization analysis, Pearson's correlation coefficient was calculated using the Coloc2 plugin in Image J to quantify the degree of colocalization between two fluorescent signals (p‐STING and CD31; STING and TBK1; NLRP3 and CASP1).

### 2.13 Transcriptome Sequencing

RNA was extracted from the samples using TRIZOL reagent according to the manufacturer's instructions. RNA quality and quantity were evaluated using a Nanodrop spectrophotometer and an Agilent Bioanalyzer. RNA libraries were prepared using the NEBNext Ultra RNA Library Prep Kit and sequenced on an Illumina platform. After filtering low‐quality reads from the raw sequencing data, the clean reads were aligned to the reference genome using STAR. Gene expression was quantified using feature counts, and differential expression analysis was conducted with DESeq2, applying a p‐value threshold of < 0.05 for significance.

### 2.14 Analysis of Surface Plasmon Resonance (SPR)

The COOH chip was prepared according to the OpenSPR instrument guidelines. A PBST buffer (pH 7.4) served as the running buffer, with the flow rate capped at 150 µL/min. After stabilizing the baseline, 200 µL of isopropanol was introduced, followed by a 10‐second air purge to clear the system. Subsequently, the chip surface was activated by injecting a 1:1 mixture of EDC and NHS at a reduced flow rate of 20 µL/min. The diluted ligand was then immobilized via a 4‐min injection, after which unbound residues were washed away. A blocking buffer was applied to passivate the surface, followed by another system wash. Before sample injection, the analyte buffer was exchanged and its stability confirmed over a 5‐min interval. The analyte, prepared at an experimentally determined concentration, was injected at 20 µL/min for 240 s (association) and 360 s (dissociation). Sensorgram data were processed and fitted using the One‐to‐One binding model in TraceDrawer software.

### 2.15 Flow Cytometry Assays

Cells were collected and suspended following the reagent kit's instructions for sample preparation. Samples were analyzed using a flow cytometer, which sorted cells into distinct populations by light scattering and fluorescence intensity, facilitating the assessment of intracellular reactive oxygen species (ROS) levels. Cells were stained with Annexin V‐FITC and PI for apoptosis detection using Annexin V‐FITC/PI. Annexin V‐FITC binds to phosphatidylserine on the surface of apoptotic cells, emitting green fluorescence. PI stains the nuclei of necrotic or late‐apoptotic cells, emitting red fluorescence. After loading onto the flow cytometer, fluorescence signals were analyzed to distinguish between normal, apoptotic, and necrotic cells. Gating strategy: All flow cytometry analyses were performed using an Agilent NOVOCYTE flow cytometer with automatic compensation functionality. Samples were prepared according to the Annexin V‐FITC/PI kit manufacturer's instructions, including negative controls (unstained cells) and single‐positive controls (Annexin V‐only and PI‐only stained cells). The sequential gating strategy was as follows: Gate 1 (Single cells): A scatter plot of FSC‐A versus SSC‐A was created to exclude debris and cell aggregates, gating on the single cell population. Gate 2 (Quadrant gates): From the single‐cell gate, a new scatter plot was generated with Annexin V‐FITC (FITC‐A) on the x‐axis and PI (PE‐A) on the y‐axis. Quadrant gates were established based on negative and single‐positive controls: (i) the horizontal threshold was set at the upper boundary of the Annexin V‐only stained population. (ii) the vertical threshold was set at the rightmost boundary of the PI‐only stained population. Double‐positive samples were used to verify appropriate quadrant placement.

### 2.16 Molecular Dynamics

This study predicted the binding modes of TBK1/STING complexes with LEO using AlphaFold Server. Mutant proteins were generated in PyMOL. LEO's structure was energy‐minimized. Molecular docking was performed with AutoDock Vina (exhaustiveness = 32) within a 25 Å^3^ box, followed by complex preparation and visualization in PyMOL. Subsequent all‐atom molecular dynamics (MD) simulations were conducted for 100 ns using AMBER 22. Ligand charges were derived with Gaussian 09, employing the GAFF2 and ff14SB force fields. Systems were solvated, neutralized, and underwent minimization, heating, and equilibration. Production runs used an NPT ensemble (298 K, 1 atm) with a 2 fs timestep. Binding free energies were calculated via the MM/GBSA (Molecular Mechanics/Generalized Born Surface Area) method on trajectories from 90 to 100 ns, utilizing the GB model (igb = 2) for polar solvation and SASA for non‐polar contributions. Entropy contributions were not included.

### 2.17 Cellular Thermal Shift Assay (CETSA)

CETSA is a powerful method for examining protein‐ligand interactions within a cellular environment. In this technique, cells or cell lysates are exposed to a gradient of temperatures, causing proteins to denature and aggregate to varying degrees based on their thermal stability. Following thermal treatment, samples are centrifuged to separate soluble (intact or ligand‐stabilized) proteins from insoluble ones. The soluble fractions are then analyzed, typically by Western blotting with specific antibodies, to detect the target protein. Proteins that bind to ligands exhibit enhanced thermal stability, remaining soluble at higher temperatures compared to their unbound forms. By plotting the protein's solubility against temperature, a melting curve is generated, revealing shifts in melting temperature upon ligand binding. CETSA thus provides direct evidence of protein‐ligand interactions, aiding in drug discovery and understanding molecular mechanisms.

### 2.18 Genotyping

WT and STING‐KO mice on a C57BL/6 background were used in this study. To confirm the knockout efficiency, cardiac tissue lysates were collected from both WT and STING‐KO mice following euthanasia. Proteins were extracted using RIPA buffer supplemented with protease inhibitors, and equal amounts of protein (30 µg per lane) were separated by SDS‐PAGE and transferred onto PVDF membranes. Membranes were incubated overnight at 4°C with anti‐STING antibody (1:1000, Cell Signaling Technology) and anti‐GAPDH antibody (1:5000, Cell Signaling Technology) as a loading control. After incubation with HRP‐conjugated secondary antibodies, protein bands were visualized using ECL kits.

### 2.19 Statistical Analysis

Figures display values as mean ± standard error of the mean (SEM). Data analysis was conducted using GraphPad Prism Version 11.0 (GraphPad Software, La Jolla, CA, USA), employing one‐way ANOVA with subsequent LSD or Dunnett's multiple comparisons tests. A *p*‐value below 0.05 was considered statistically significant.

## 3 Results

### 3.1 LEO Improves Cardiac Function in DIC Mice

**FIGURE 1:**
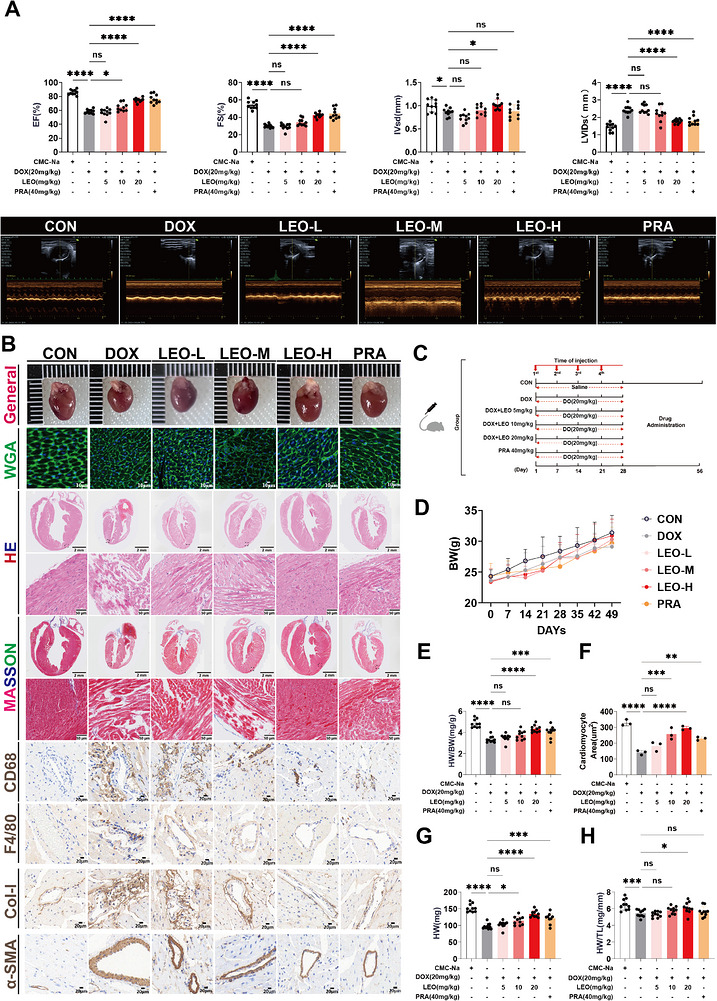
LEO ameliorates cardiac function in DIC mice. (A) Evaluation of cardiac function (n = 10). (B) Gross view of the heart, WGA, HE, MASSON staining, as well as immunohistochemistry for CD68, F4/80, COL‐I, and α‐SMA (n = 3). (C) Establishment of the model and treatment regimen. (D‐H) Body weight (BW)‐time curve, heart mass (HW), general heart area, heart mass‐to‐body mass ratio (HW/BW), and heart mass‐to‐tibia length ratio (HW/TL) in mice (n = 3). ^*^ *p* < 0.05, ^**^ *p* < 0.01, ^***^ *p* < 0.001, ^****^ *p* < 0.01 and ns: Not significant.

The mouse breeding and intervention protocols were conducted in accordance with the established plan (Figure 1C). Echocardiography results demonstrated that LEO could enhance cardiac ejection fraction (EF), fractional shortening (FS), and interventricular septal thickness at diastole (IVsd), while reducing left ventricular internal diameter at systole (LVIDs) in DIC mice (Figure 1A). Additionally, LEO decreased heart mass, gross heart area, heart mass‐to‐body mass ratio, and heart mass‐to‐tibia length ratio, while increasing body mass in DIC mice (Figure 1B,D–H). Survival analysis revealed that LEO significantly improved the survival prognosis of DIC mice (Figure S2). WGA staining results indicated that LEO notably increased cardiomyocyte area in DIC mice, and PCR results showed that LEO decreased the mRNA levels of cardiac ANP, BNP, β‐MHC, related to myocardial cytoskeletal structure (Figure 1B, Figure S15B). HE staining, along with CD68 and F4/80 immunohistochemical staining, revealed disorganized myocardial arrangement, severe perivascular pathological damage, and increased inflammatory cell infiltration in DIC mice, all of which were markedly improved following LEO treatment (Figure 1B, Figure S15C). MASSON staining, immunohistochemistry, and PCR results demonstrated that LEO significantly reduced cardiac collagen deposition (Figure 1B, Figure S1A and S15A,D). Serological ELISA tests found that LEO decreased serum levels of CKMB, LDH, Myo, cTNT, and NT‐proBNP in DIC mice (Figure S15E). Considering that DIC primarily manifests as myocardial mitochondrial damage, transmission electron microscopy revealed that LEO alleviated myocardial sarcomere disorganization and mitochondrial damage, and also reduced lysosomal accumulation in DIC mice (Figure S1B). Our team's previous research work has confirmed that within 1 to 4 weeks of DIC progression, although cardiac autophagy is activated, the autophagy flow is actually impaired, and the degree of autophagy blockage is significantly positively correlated with the severity of mitochondrial ultrastructural damage and the decline in cardiac function [27]. PCR results also showed that LEO treatment upregulated the mRNA levels of PINK and PARKIN, while downregulating DRP1 (Figure S1C). Furthermore, the above results indicated that the pharmacological effects of LEO exhibited a certain dose‐dependent pattern.

### 3.2 LEO Suppresses the cGAS/STING/NF‐κB/NLRP3 Signaling Pathway in Cardiac Endothelial Cells of DIC Mice

**FIGURE 2:**
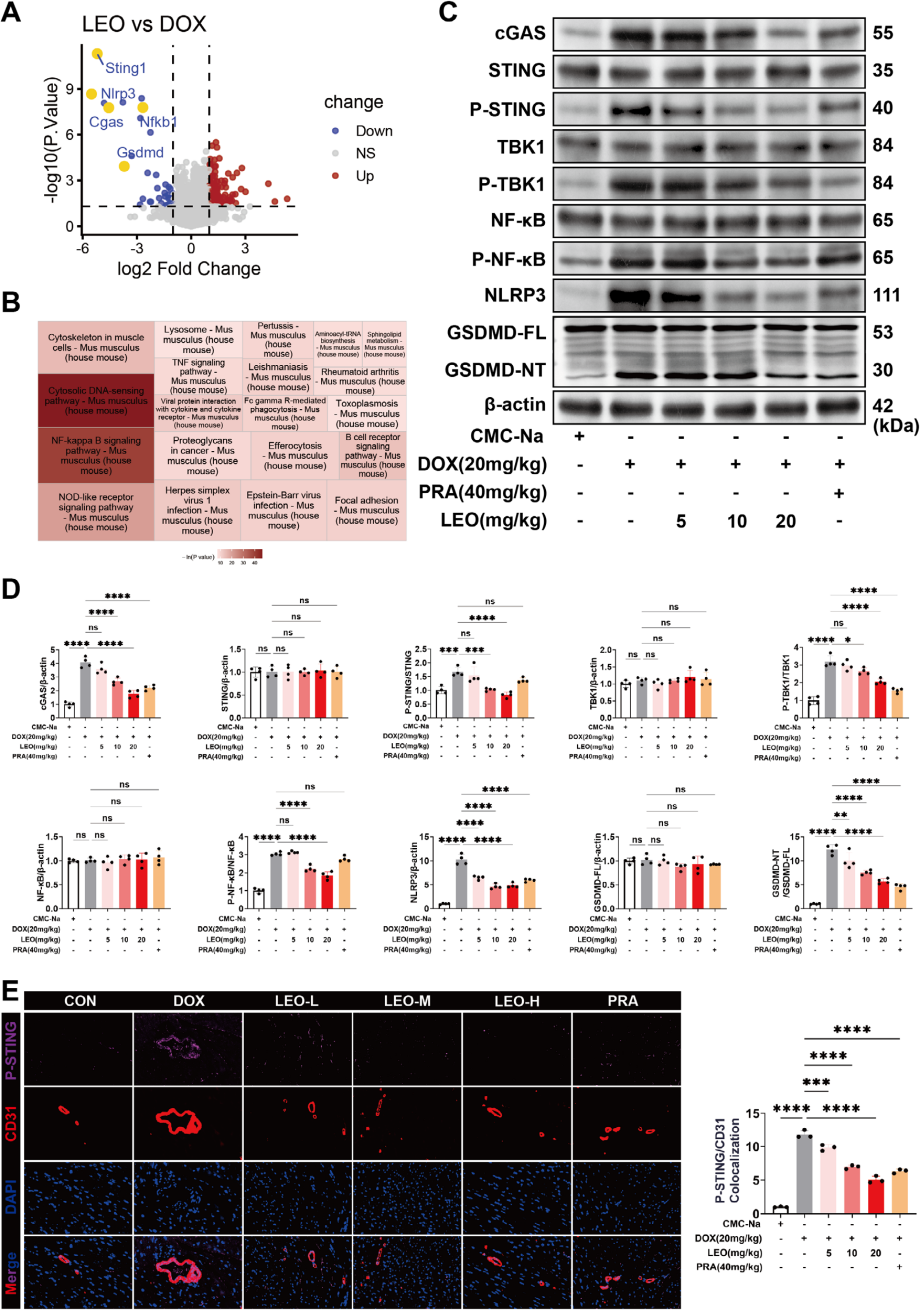
LEO downregulates the cGAS/STING/NF‐κB/NLRP3 pathway in cardiac endothelial cells of DIC mice. (A) Volcano plot of differentially expressed genes between the DOX group and the LEO group (n = 3). (B) KEGG enrichment analysis of differentially expressed genes between the DOX group and the LEO group (n = 3). (C & D) Protein expression levels of cGAS, p‐STING/STING, p‐TBK1/TBK1, p‐NF‐κB/NF‐κB, NLRP3, and GSDMD‐FL/GSDMD‐NT in the hearts of mice (n = 3). (E) Immunofluorescence and statistical analysis of P‐STING and CD31 in the hearts of DIC mice (n = 3). ^*^ *p* < 0.05, ^**^ *p* < 0.01, ^***^ *p* < 0.001, ^****^ *p* < 0.01 and ns: Not significant.

Through transcriptomic studies, PCA results revealed significant alterations in the distribution of cardiac transcripts among the CON, DOX, and LEO groups of mice (Figure S3A). Compared to the CON group, genes such as STING1, cGAS, NF‐κB, NLRP3, and GSDMD were markedly upregulated in the hearts of DOX‐treated mice (Figure S3B). However, it was found that LEO could significantly downregulate genes including STING1, TBK1, cGAS, NF‐κB, NLRP3, CASP1, ASC, and GSDMD in the hearts of DIC mice (Figure 2A, Figure S16A). Gene Ontology (GO) enrichment analysis indicated that LEO affected the transcriptional factor activities related to the Golgi membrane, immune response, and extracellular vesicles in the hearts of DIC mice (Figure S3C,D). Meanwhile, Kyoto Encyclopedia of Genes and Genomes (KEGG) results showed that the differentially expressed genes between the LEO and DOX groups were primarily enriched in pathways such as the Cytosolic DNA‐sensing pathway, NF‐κB signaling pathway, and NOD‐like receptor signaling pathway, which were similar to the enrichment results observed between the DOX and CON groups (Figure 2B, Figure S3E,F). The above findings suggest that the improving effect of LEO on the hearts of DIC mice may be associated with the cGAS/STING/NF‐κB/NLRP3 pathway. Further, WB results demonstrated that LEO could reduce the protein levels of cGAS, p‐STING/STING, p‐TBK1/TBK1, p‐NF‐κB/NF‐κB, NLRP3, GSDMD‐FL/GSDMD‐NT in the hearts of DIC mice (Figure 2C,D). RT‐qPCR results showed that LEO could decrease the mRNA levels of cGAS, STING, TBK1, IKKα, NF‐κB, ASC, NLRP3, GSDMD, CASP1, IL18, IL1β in the hearts of DIC mice (Figure S16B). Considering that NLRP3 inflammasome activation primarily mediates inflammatory responses, we detected serum levels of IL18, IL1β, TNFα, ICAM‐1, ICAM‐2, and IL10 via ELISA and found that LEO indeed improved the levels of these inflammatory factors (Figure S16C). The above results indicate that LEO treatment indeed downregulates the cGAS/STING/NF‐κB/NLRP3 pathway in the hearts of DIC mice. However, in which type of cells does this process primarily occur? We then employed immunofluorescence and found that the levels of p‐STING, as well as its colocalization with CD31, were significantly elevated in cardiac vascular endothelial cells of DIC mice, whereas these levels were markedly reduced following LEO treatment (Figure 2E, Figure S16D). Subsequently, through transmission electron microscopy, we observed that the LEO group significantly enhanced the tight junctions of cardiac vascular endothelium and markedly alleviated the collapse of the vascular basement membrane (Figure S4A). So, how is the cGAS/STING signaling pathway activated in endothelial cells? Considering that previous studies have shown that DOX directly induces myocardial mitochondrial damage, a process that may release mtDNA and thereby activate cGAS. We confirmed via PCR that the release of mtDNA such as MT‐CO1, MT‐CO2, and MT‐ATP6 in the circulation was significantly increased in DIC, whereas LEO treatment markedly reduced it (Figure S4B).

### 3.3 LEO Mitigates DOX‐Induced Injury in Cardiac Vascular Endothelial Cells and Suppresses the cGAS/STING/NF‐κB/NLRP3 Signaling Pathway

**FIGURE 3:**
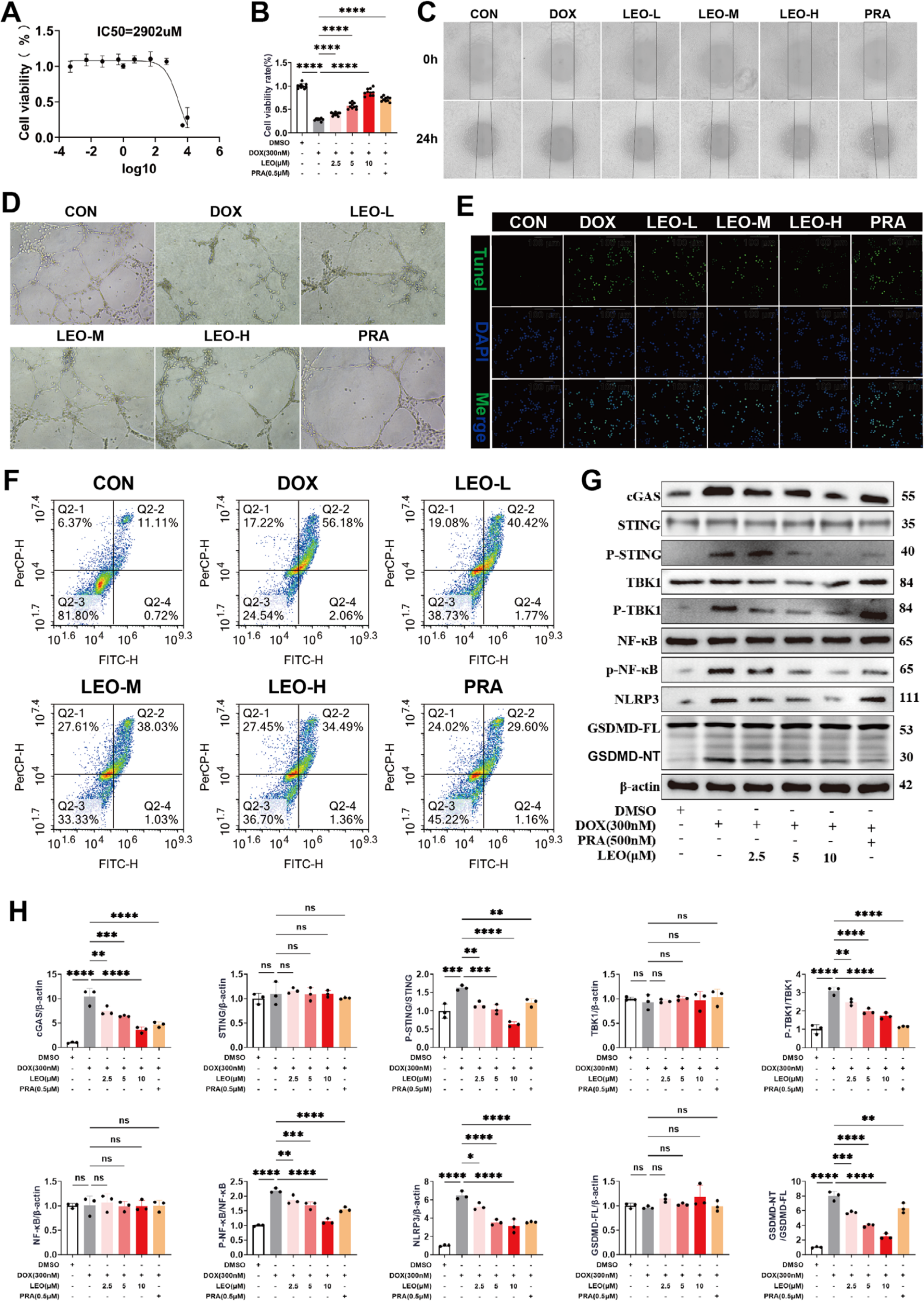
LEO ameliorates DOX‐mediated cardiac vascular endothelial cell (CMVEC) injury and downregulates the cGAS/STING/NF‐κB/NLRP3 pathway. (A) IC50 of LEO on CMVEC. (B) Effects of LEO on CMVEC viability (n = 3). (C) Wound healing assay of CMVEC treated with LEO (n = 3). (D) Tube formation assay of CMVEC treated with LEO. (E) TUNEL staining and statistical analysis. (F) Annexin V‐FITC/PI flow cytometry analysis of CMVEC treated with LEO. (G, H) WB experiments and statistical analysis of cGAS, p‐STING/STING, p‐TBK1/TBK1, p‐NF‐κB/NF‐κB, NLRP3, and GSDMD‐FL/GSDMD‐NT. ^*^ *p* < 0.05, ^**^ *p* < 0.01, ^***^ *p* < 0.001, ^****^ *p* < 0.01 and ns: Not significant.

We conducted a series of experiments using CMVEC/HUVEC cells with STING overexpression/knockdown. After determining the IC50 values of LEO for CMVEC/HUVEC as 2902 µM / 2241 µM, we preliminarily established an equivalent dose of 5 µM for LEO (Figure 3A, Figure S8A). CCK8 assays demonstrated that LEO enhanced cell survival in CMVEC/HUVEC cells under DOX intervention (Figure 3B, Figure S8B). The wound healing assay revealed that LEO improved the cell migration rate of CMVEC/HUVEC cells under DOX intervention, which is crucial for vascular barrier function (Figure 3C, Figures S8C,F, and S17A). The tube formation assay showed that LEO enhanced the angiogenic capacity of CMVEC/HUVEC cells under DOX intervention, which is significant for compensatory angiogenesis in DIC hearts (Figure 3D, Figures S8D,G and S17B). Tunel staining demonstrated that LEO decreased the apoptosis rate of CMVEC/HUVEC cells induced by DOX (Figure 3E, Figures S8E,H and S17D). Annexin V‐FITC/PI flow cytometry indicated that LEO reduced DOX‐induced necrosis in CMVEC cells (Figure 3F, Figure S17C). Further, WB results showed that LEO downregulated the protein levels of cGAS, p‐STING/STING, p‐TBK1/TBK1, p‐NF‐κB/NF‐κB, NLRP3, and GSDMD‐FL/GSDMD‐NT in CMVEC cells (Figure 3G, H). RT‐qPCR results indicated that LEO reduced the mRNA levels of cGAS, STING, TBK1, IKKα, NF‐κB, ASC, NLRP3, GSDMD, CASP1, IL18, and IL1β in CMVEC/HUVEC cells (Figures S8I and S17E). ELISA results showed that LEO decreased the release of inflammatory factors such as IL18, IL1β, TNFα, ICAM‐1, ICAM‐2, and CASP1 in CMVEC/HUVEC cells (Figures S8J and S17F). We also tracked the entire activation process of the cGAS/STING/NF‐κB/NLRP3 pathway using immunofluorescence and found that LEO reduced the levels of STING, TBK1, STING/TBK1 colocalization, NF‐κB, NF‐κB nuclear‐cytoplasmic ratio, NLRP3, CASP1, and NLRP3/CASP1 colocalization (Figure S5A‐D). To further investigate whether the pharmacological improvement effect of LEO depends on STING, we found through STING overexpression that STING overexpression completely reversed the therapeutic effect of LEO, while STING knockdown improved DOX‐induced cellular damage and showed no additive effect with LEO treatment (Figures S6, S7, and S8K,L).

### 3.4 LEO Binds to the STING Protein via the TYR261 Residue

**FIGURE 4:**
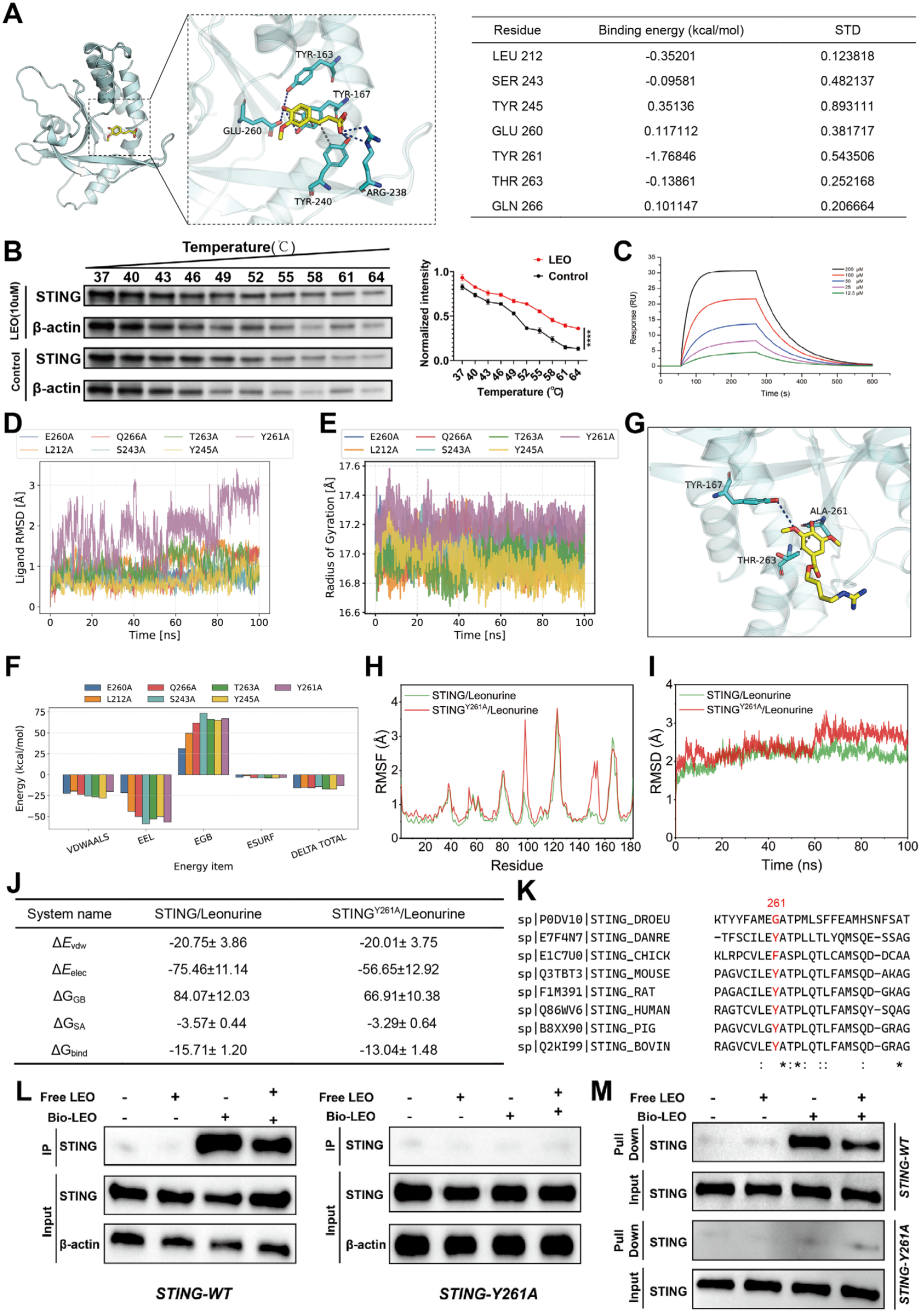
LEO binds to the STING protein via the TYR261 amino acid residue. (A) 3D conformational diagram of molecular docking between LEO and human STING protein, along with the binding affinity of relevant residues. (B) CETSA results for LEO binding to STING. (C) SPR results for LEO binding to human STING protein. (D) Molecular dynamics simulation of Ligand RMSD for LEO and the STING protein mutant. (E) Molecular dynamics simulation of the RoG for LEO and the STING protein mutant. (F) Molecular dynamics simulation of MM‐GBSA for LEO and the STING protein mutant. (G) 3D diagram of molecular docking between STING^TYR261A^ and LEO. (H) Molecular dynamics simulation of RMSD for STING^TYR261A^ and LEO. (I) Molecular dynamics simulation of RMSF (Root Mean Square Fluctuation) for STING^TYR261A^ and LEO. (J) Molecular dynamics simulation of MM‐GBSA for STING^TYR261A^ and LEO. (K) Amino acid sequence alignment of the STING TYR261 site across multiple species. (L) CO‐IP results for Bio‐LEO with STING^WT^/STING^TYR261A^. (M) Pull‐down assay results for Bio‐LEO with human STING/STING^TYR261A^ protein. ^*^ *p* < 0.05, ^**^ *p* < 0.01, ^***^ *p* < 0.001, ^****^ *p* < 0.01 and ns: Not significant.

After investigating the impact of LEO on the cGAS/STING/NF‐κB/NLRP3 pathway in DIC cardiac endothelial cells, it became crucial to study the structural biological mechanism underlying LEO's binding to the STING protein. Initially, through molecular docking, we discovered that LEO could bind effectively to the human STING protein, with the primary binding sites being LEU212, SER243, TYR245, GLU260, TYR261, THR263, and GLN266. Among these, TYR261 received the highest score (Figure 4A). Furthermore, our CETSA experiments revealed that LEO enhanced the thermal stability of the STING protein within CMVEC (Figure 4B). We also expressed and purified the human STING protein, and through SPR experiments, we found that LEO exhibited a good affinity for the human STING protein (KD(M) = 7.18E‐05), which falls within the effective binding range for small‐molecule traditional Chinese medicine compounds interacting with target proteins (Figure 4C and Table S2). Further, we created point‐mutated STING proteins and found via simulations that only the TYR261 mutation increased small‐molecule motion in the binding pocket, causing unstable binding that might dissociate or adjust. This indicates Y261 is key for small‐molecule positioning (Figure 4D). The radius of gyration (RoG) showed the Y261A complex was consistently looser than others, starting at ∼17.4–17.5 Å and staying around 17.2–17.4 Å, indicating instability (Figure 4E). Using MM‐GBSA on simulation trajectories, we calculated binding energies. The E260A, L212A, Q266A, S243A, T263A, Y245A, and Y261A complexes had energies of −15.83 ± 1.11, −15.07 ± 1.44, −15.50 ± 1.13, −14.23 ± 0.88, −17.30 ± 1.13, −17.28 ± 1.10, and −13.05 ± 1.49 kcal/mol, respectively. Negative values indicate binding affinity, with lower values meaning stronger binding. The Y261A mutant showed the weakest binding, highlighting Y261 as crucial (Figure 4F, Figure S9). To rule out chance, we repeated the TYR261 mutation simulation. The docking showed LEO formed hydrogen bonds with TYR‐167 and ALA‐261 in STINGY261A, plus hydrophobic interactions with THR‐263. Compared to the wild‐type, the mutant's binding mode changed significantly, with fewer binding sites (Figure 4G). Root‐mean‐square deviation (RMSD) from simulations showed the STING/Leonurine complex stabilized early, fluctuating within 0–3 Å, indicating stability. The mutant (green line) fluctuated more than the wild‐type (red line), showing poorer binding stability (Figure 4H). Root‐mean‐square fluctuation (RMSF) revealed that except for local regions, the protein's main structure was rigid (<2 Å). The mutant had higher RMSF, meaning poorer inhibition by the small molecule (Figure 4I). MM‐GBSA ((Molecular Mechanics‐Generalized Born Surface Area)) showed binding energies of −15.71±1.20 for STING/Leonurine and −13.04 ± 1.48 for STINGY261A/Leonurine. Negative values indicate affinity, with STING/Leonurine binding stronger. Energy decomposition showed electrostatic and van der Waals forces were the main contributors, followed by non‐polar solvation energy (Figure 4J). To rule out the impact of amino acid site variations in multi‐species proteins, we examined the STING protein's residue 261 across multiple species through data analysis. We found that TYR261 is highly conserved during evolution (especially in mammals such as humans, rats, mice, and pigs), and several adjacent sites also exhibit high conservation, providing sequence support for the stability of the molecular structural domain (Figure 4K).

Subsequently, we conducted intracellular CO‐IP experiments for validation. We found that Bio‐LEO could effectively pull down the STING protein, and this effect was competitively inhibited by Free LEO. Moreover, the TYR261 mutation abolished this pull‐down effect (Figure 4L). Similar results were obtained through pulldown assays using purified human STING protein. Specifically, Bio‐LEO could effectively pull down the STING protein, and this effect was competitively inhibited by Free LEO. Additionally, the TYR261 mutation abolished this pull‐down effect (Figure 4M).

### 3.5 LEO Inhibits the Oligomerization of STING Protein and the Formation of the STING/TBK1 Protein Heterodimer

**FIGURE 5:**
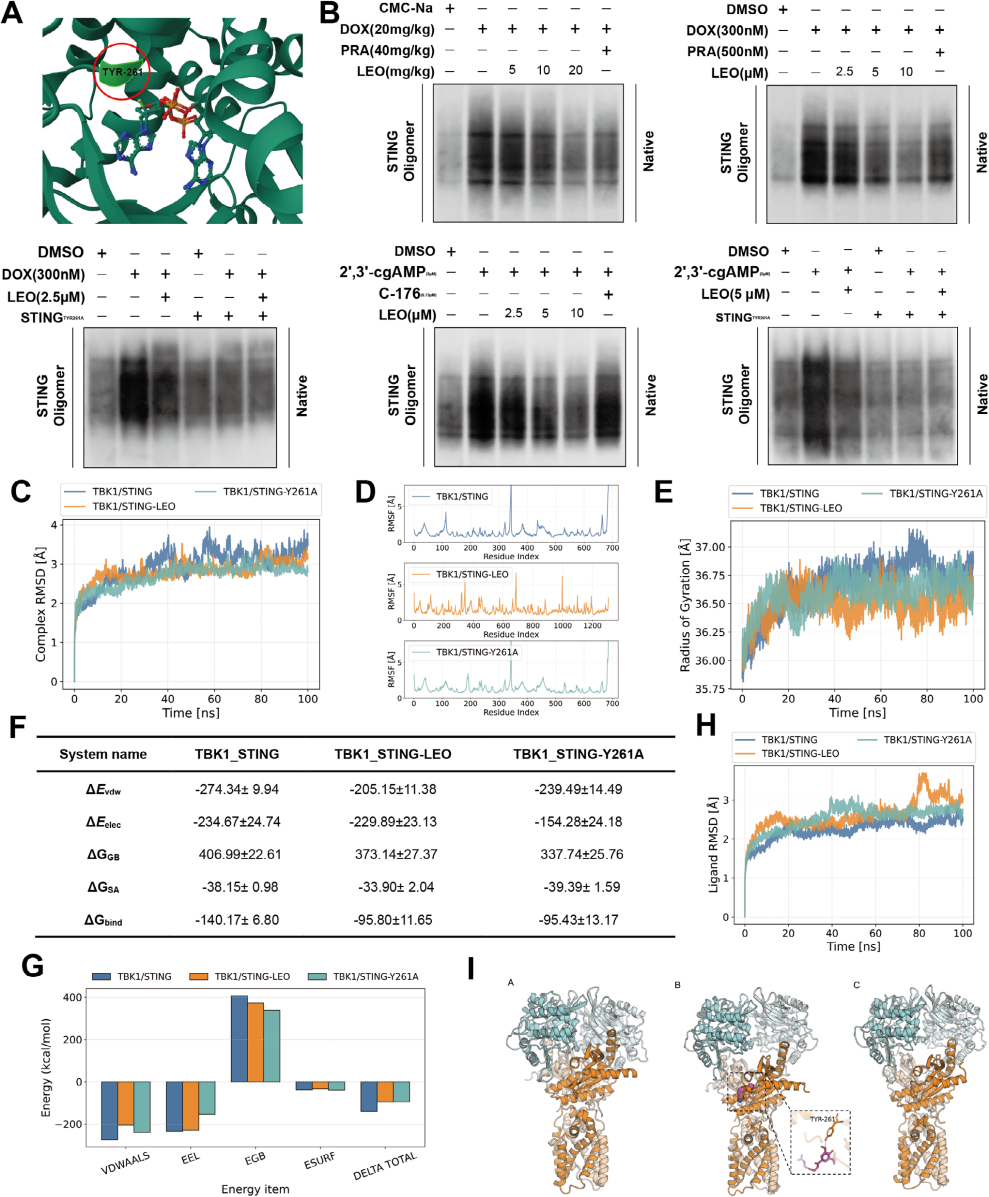
LEO inhibits the oligomerization of the STING protein and the formation of the STING/TBK1 protein heterodimer. (A) Position of TYR‐261 in the STING dimer. (B) In vitro and in vivo WB experiments examining the effect of LEO on the oligomerization of STING/STING^TYR261A^. (C) Molecular dynamics simulation of RMSD for LEO and the human STING protein. (D) Molecular dynamics simulation of RMSF for LEO and the human STING protein. (E) Molecular dynamics simulation of ROG for LEO and the human STING protein. (F, G) Molecular dynamics simulation of MM‐GBSA for LEO and the human STING protein. (H) Molecular dynamics simulation of RMSD for LEO and the STING/TBK1 dimer. (I) 3D diagrams of molecular docking between LEO and the STING/TBK1 dimer. A: Native docking structure of wild‐type STING and TBK1; B: Docking structure of wild‐type STING and TBK1 in the presence of LEO; C: Docking structure of STING^TYR261A^ and wild‐type TBK1 in the presence of LEO. ^*^ *p* < 0.05, ^**^ *p* < 0.01, ^***^ *p* < 0.001, ^****^ *p* < 0.01 and ns: Not significant.

Previous studies published in Nature and Cell have demonstrated that upon induction by 2',3'‐cGAMP derived from cGAS, STING undergoes translocation from the endoplasmic reticulum to the Golgi [28]. During this process, STING oligomerizes, and each STING protein forms a dimer with a TBK1 protein [29, 30]. This misaligned activation prompts the sequential phosphorylation of TBK1 and STING, ultimately activating the downstream NF‐κB signaling pathway—a process crucial for STING signal activation [31]. In our aforementioned research, we have found that LEO can reduce the phosphorylation levels of STING and TBK1, as well as their co‐localization. However, the specific mechanism remained unclear. Through structural analysis of the STING protein oligomer, we discovered that TYR261 is located at the central position of the STING protein binding interface and is adjacent to the molecular pocket (Figure 5A). We hypothesize that the competitive binding of LEO to the TYR261 site may destabilize the STING protein oligomer. WB confirmed that LEO indeed reduces STING protein oligomerization levels in the DIC mice, as well as in CMVEC subjected to DOX/2'3'‐cGAMP treatment or harboring point mutations. Moreover, mutation at the TYR261 site similarly decreases the oligomerization level of the STING protein, with no additive effect observed when combined with LEO (Figure 5B). As mentioned above, LEO inhibits the oligomerization of the STING protein and may subsequently suppress the formation of the STING/TBK1 heterodimer, though further confirmation is needed. We constructed binding models of LEO, TBK1, STING, and STING^TYR261A^ using molecular dynamics simulations. RMSD analysis showed that all three systems rapidly reached stability, with the LEO‐bound and Y261A‐mutated systems exhibiting lower RMSD and narrower fluctuation ranges compared to the wild‐type, indicating restricted conformational adjustments between TBK1 and STING (Figure 5C). Thus, both LEO binding and the Y261A mutation are unfavorable for TBK1/STING complex polymerization. The RMSF curve of the wild‐type complex revealed significant flexibility in the activation loop (residues 350–380) and C‐terminal tail (residues ≈660–700). LEO binding and the Y261A mutation reduced fluctuations in the activation loop, though the C‐terminal tail remained highly flexible (Figure 5D). Radius of gyration (Rg) analysis demonstrated that LEO binding significantly enhanced compactness and structural stability, while the Y261A mutation had a moderate effect, both reducing overall expansion compared to the wild‐type (Figure 5E). MM‐GBSA calculations showed that binding energies for TBK1_STING, TBK1_STING‐LEO, and TBK1_STING‐Y261A were −140.17 ± 6.80, −95.80 ± 11.65, and −95.43 ± 13.17 kcal/mol, respectively, indicating weaker binding and reduced polymerization upon LEO binding or Y261A mutation (Figure 5F,G). Ligand RMSD suggested instability in the STING/TBK1 heterodimer with LEO or Y261A (Figure 5H). Typical conformations showed that LEO binding or Y261A mutation disrupted the interface between STING and TBK1, weakening C‐terminal tail adhesion and hindering polymerization (Figure 5I).

### 3.6 LEO Reduces the 2',3'‐cGAMP‐Induced Specific Activation of the STING/NLRP3 Signaling Pathway in Vascular Endothelial Cells

**FIGURE 6:**
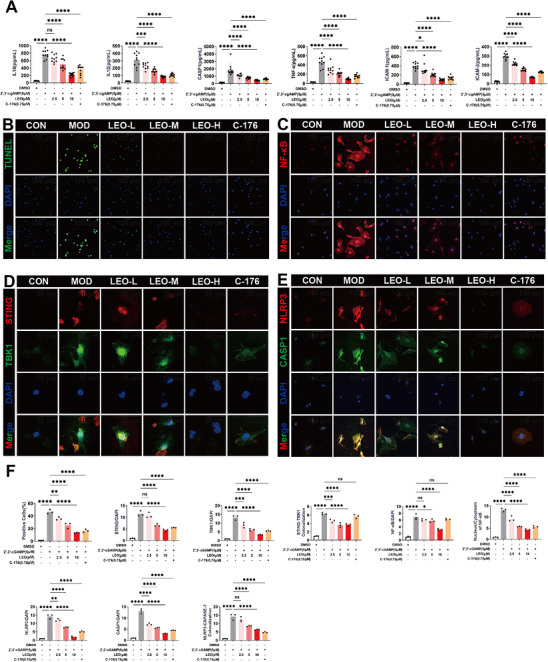
LEO reduces the 2',3'‐cGAMP‐induced specific activation of the STING/NLRP3 signaling pathway in CMVEC. (A) Levels of IL18, IL1β, TNFα, ICAM‐1, ICAM‐2, and CASP1 in the culture supernatant (n = 10). (B) TUNEL staining (n = 3). (C–E) Immunofluorescence staining of STING, TBK1, co‐localization of STING/TBK1, NLRP3, CASP1, co‐localization of NLRP3/CASP1, and NF‐κB (n = 3). (F) Statistical analysis of IF and Tunel staining results (n = 3). ^*^ *p* < 0.05, ^**^ *p* < 0.01, ^***^ *p* < 0.001, ^****^ *p* < 0.01 and ns: Not significant.

To further elucidate the mechanism by which LEO targets the STING protein to inhibit the NF‐κB/NLRP3/GSDMD signaling pathway, we established a model of STING‐specific activation in CMVEC/HUVEC cells by administering exogenous 2',3'‐cGAMP. ELISA results demonstrated that LEO significantly reduced the release of cytokines such as IL18, IL1β, TNFα, ICAM‐1, ICAM‐2, and CASP1 in CMVEC/HUVEC cells (Figure 6A, Figure S12C). Tunel assay results showed that LEO decreased the positivity rate in CMVEC/HUVEC cells triggered by STING signal activation (Figure 6B,F, Figure S12A). RT‐qPCR results indicated that LEO reduced the mRNA levels of cGAS, STING, TBK1, IKKα, NF‐κB, NLRP3, GSDMD, and CASP1 in CMVEC/HUVEC cells (Figures S12B and S18). IF results demonstrated that LEO reduced the levels of STING, TBK1, STING/TBK1 co‐localization, NLRP3, CASP1, NLRP3/CASP1 co‐localization, NF‐κB, and the nuclear‐cytoplasmic ratio of NF‐κB in CMVEC/HUVEC (Figure 6C–F). To further investigate whether the pharmacological improvement effect of LEO depends on STING, we found that STING overexpression completely reversed the therapeutic effect of LEO. Conversely, STING knockdown improved DOX‐induced cellular damage, and there was no additive effect with LEO treatment (Figures S10, S11, S12D,E).

### 3.7 LEO Inhibits Mitochondrial Dysfunction in Cardiomyocytes Induced by Pyroptosis‐Derived EVs From Endothelial Cells via Targeting the STING /GSDMD Pathway

**FIGURE 7:**
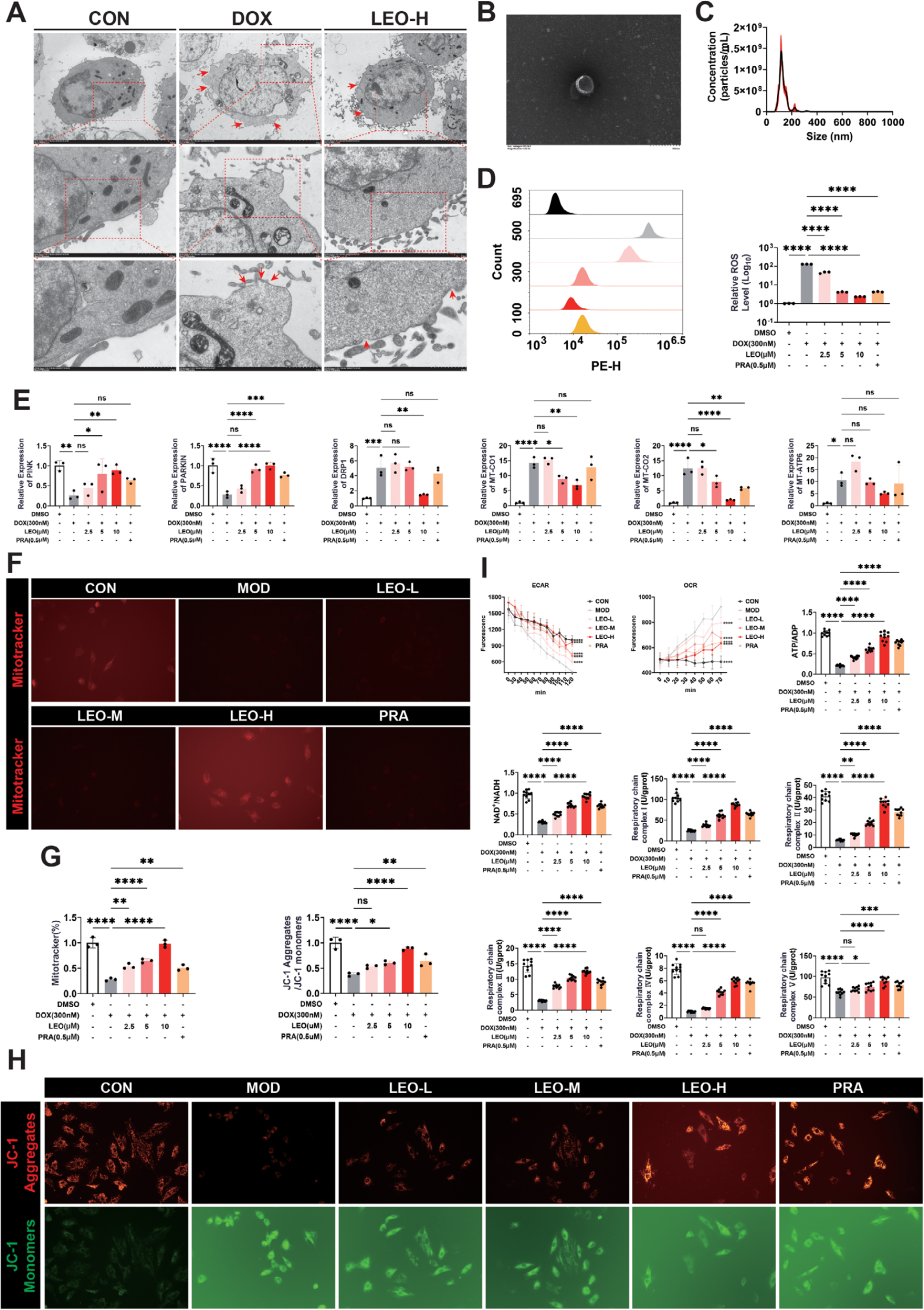
LEO inhibits mitochondrial dysfunction in cardiomyocytes induced by pyroptosis‐derived EVs from endothelial cells via targeting the STING/GSDMD pathway. (A) Transmission electron microscopy (TEM) results of CMVEC. Arrows indicate cellular holes or membrane defects (n = 3). (B) TEM examination of EVs in the supernatant of CMVEC. (C) NTA of vesicles in the supernatant of CMVEC. (D) Detection of the effects of vesicle transplantation on cardiomyocytes by ROS assay (n = 3). (E) PCR analysis of PINK, PARKIN, DRP1, MT‐CO1, MT‐CO2, and MT‐ATP6 levels in cardiomyocytes (n = 3). (F–H) JC‐1 staining, Mitotracker staining, and statistical analysis of cardiomyocytes (n = 3). (I) Detection of ECAR, OCR, ATP/ADP ratio, NAD+/NADH ratio, and mitochondrial respiratory chain complex (I‐V) activity in cardiomyocytes (n = 10). ^*^ *p* < 0.05, ^**^ *p* < 0.01, ^***^ *p* < 0.001, ^****^ *p* < 0.01 and ns: Not significant.

The aforementioned studies have largely confirmed that LEO reduces pathological damage by inhibiting the STING/NLRP3/GSDMD pathway in endothelial cells. However, the relationship between this and the widespread mitochondrial damage observed in cardiomyocytes in the context of DIC remains to be elucidated. In our previous studies, we obtained evidence suggesting that pyroptosis‐derived EVs can induce “bystander” cell damage or even death. However, this mechanism has not been clarified between cardiac endothelial cells and cardiomyocytes in DIC. Considering that the STING/NLRP3/GSDMD pathway primarily mediates pyroptosis and that DIC cardiomyocytes predominantly exhibit mitochondrial damage, leading to substantial release of mtDNA, a crucial signaling molecule for activating the cGAS/STING pathway. We questioned whether pyroptosis‐derived EVs play a significant role in the crosstalk between DIC endothelial cells and cardiomyocytes. We further conducted a series of experiments. First, using scanning electron microscopy, we observed that endothelial cells exhibited a pyroptotic phenotype and released “EVs”‐like structures under DOX intervention, whereas LEO treatment improved pyroptosis and significantly reduced the release of these “EVs”‐like structures (Figure 7A). Furthermore, we successfully extracted vesicles from the supernatant of endothelial cells after DOX intervention. Electron microscopy revealed that these EVs possessed a distinct double‐layered membrane structure, and NTA analysis indicated that their particle size range was appropriate (Figure 7B,C). We then investigated whether these EVs could affect cardiomyocyte mitochondria. By extracting extracellular EVs from different groups and reinfusing them into cardiomyocytes, we found that EVs from the DOX group significantly elevated ROS levels in cardiomyocytes, whereas the LEO group showed continuous improvement in ROS levels with increasing doses (Figure 7D). PCR results demonstrated that the DOX group exhibited significantly reduced mRNA levels of PINK and PARKIN in cardiomyocytes, along with elevated mRNA levels of DRP1 and mtDNA release markers such as MT‐CO1, MT‐CO2, and MT‐ATP6. In contrast, the LEO group showed significant improvement (Figure 7E). As shown in the new Figure S21, a significant accumulation of cytosolic dsDNA in the hearts of DIC mice and H9C2 was observed. Notably, treatment with LEO markedly reduced this dsDNA accumulation, further supporting its protective role upstream of the cGAS/STING cascade. This confirms the release of DNA fragments from damaged cardiomyocyte mitochondria into the cytoplasm. Additionally, JC‐1 and mitotracker staining revealed that the DOX group exhibited a marked decrease in mitochondrial membrane potential and mitochondrial number in cardiomyocytes, whereas the LEO group showed significant improvement (Figure 7F‐H). To further assess energy metabolism in cardiomyocytes, we conducted metabolomic analysis and found that LEO nearly reversed the overall metabolic pattern of cardiomyocytes, with multiple metabolite levels returning to normal. KEGG enrichment analysis revealed that LEO primarily affected the TCA (Tricarboxylic acid) Cycle (Figure S13). Based on these findings, we further examined mitochondrial energy metabolism in cardiomyocytes and found that vesicles from the DOX group significantly worsened ECAR, OCR, ATP/ADP, NAD+/NADH, and the mitochondrial respiratory chain complex (I‐V) in cardiomyocytes, whereas the LEO group showed improvement (Figure 7I). The aforementioned studies suggest that EVs derived from endothelial cell pyroptosis indeed cause mitochondrial damage in cardiomyocytes. Is there a causal relationship between the STING/GSDMD pathway and this phenomenon? We first pretreated the extracted vesicles from each group with DSF to specifically inhibit GSDMD activity and then administered them to cardiomyocytes. We found that DSF‐pretreated vesicles did not exhibit any mitochondrial toxicity to cardiomyocytes (Figure S14A‐C,H). Conversely, overexpression of STING in endothelial cells significantly enhanced the mitochondrial toxicity of the EVs (Figure S14D,E,I). However, when we administered DSF treatment again, the EVs lost their mitochondrial toxicity (Figure S14F,G,J). In conclusion, we confirmed that EVs derived from cGAS/STING signaling in cardiac vascular endothelial cells mediate mitochondrial damage in cardiomyocytes in DIC, and that LEO treatment effectively blocks this process.

Furthermore, to evaluate whether LEO could directly protect cardiomyocytes independent of its effects on endothelial cells, we treated H9C2 cardiomyocytes with DOX in the presence or absence of LEO. As shown in Figure S19, LEO directly applied to cardiomyocytes partially ameliorated DOX‐induced mitochondrial dysfunction, as evidenced by reduced LDH release and BNP expression; improved ATP/ADP and NAD^+^/NADH ratios; restored PINK/PARKIN expression; downregulated DRP1; and decreased mtDNA release (MT‐CO1, MT‐CO2, MT‐ATP6). These results demonstrate that LEO possesses intrinsic mitochondrial protective properties, complementing its primary mechanism of endothelial STING inhibition.

### 3.8 STING‐KO Improves Cardiac Function in DIC Mice and Shows no Additive Effect With LEO Treatment

**FIGURE 8:**
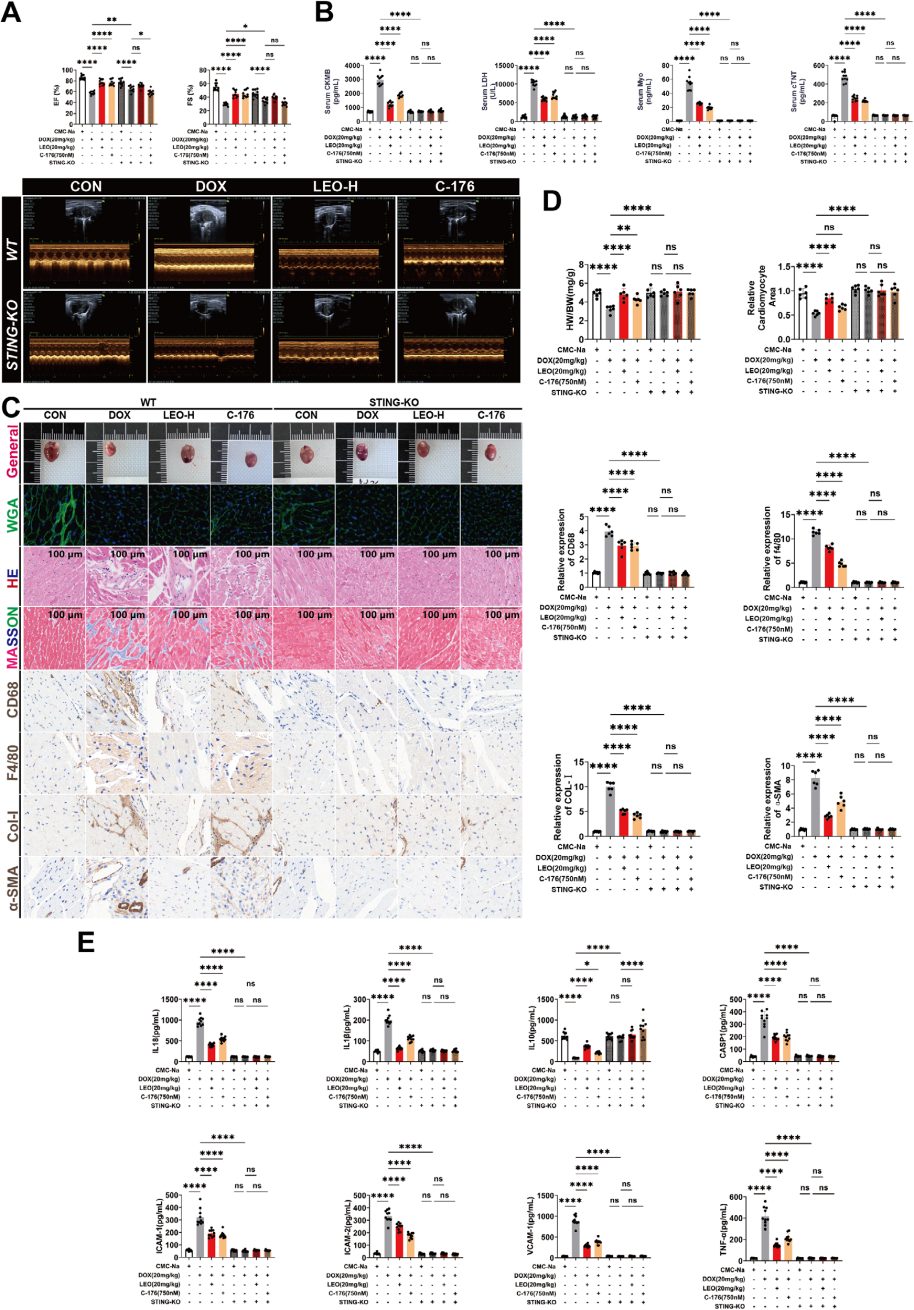
STING‐KO improves cardiac function in DIC mice and shows no additive effect with LEO treatment. (A) Assessment of cardiac function in mice. (B) Levels of CKMB, LDH, Myo and cTNT in mouse serum. (C) Gross observation of mouse hearts, along with WGA staining, HE staining, MASSON staining, and immunohistochemical examination. (D) Measurement of HW/BW, cardiomyocyte area, cardiac inflammation, and fibrosis levels. (E) Levels of serum inflammatory factors and vascular endothelial damage factors. ^*^ *p* < 0.05, ^**^ *p* < 0.01, ^***^ *p* < 0.001, ^****^ *p* < 0.01 and ns: Not significant.

To further elucidate that the therapeutic effect of LEO on DIC primarily depends on STING, we repeated the pharmacodynamic experiments using STING‐KO mice. As shown in Figure S20, STING protein expression was completely absent in STING‐KO mice compared to WT controls, confirming successful gene deletion at the protein level. Color Doppler ultrasound results indicated that STING‐KO markedly improved cardiac function in DIC mice, whereas LEO treatment did not show further overall improvement (Figure 8A). ELISA results demonstrated that STING‐KO significantly improved the serum levels of CKMB, LDH, Myo and cTNT in DIC mice (Figure 8B). Furthermore, STING‐KO significantly improved mouse body weight, heart mass, heart‐to‐tibia ratio, cardiomyocyte area, cardiac inflammation, and fibrosis levels, while LEO treatment did not demonstrate further improvement (Figure 8C,D). Additionally, STING‐KO significantly improved the serum levels of IL18, IL1β, IL10, CASP1, ICAM‐1, ICAM‐2, VCAM‐1, and VCAM‐2 in mice (Figure 8E).

## 4 Discussion

**FIGURE 9:**
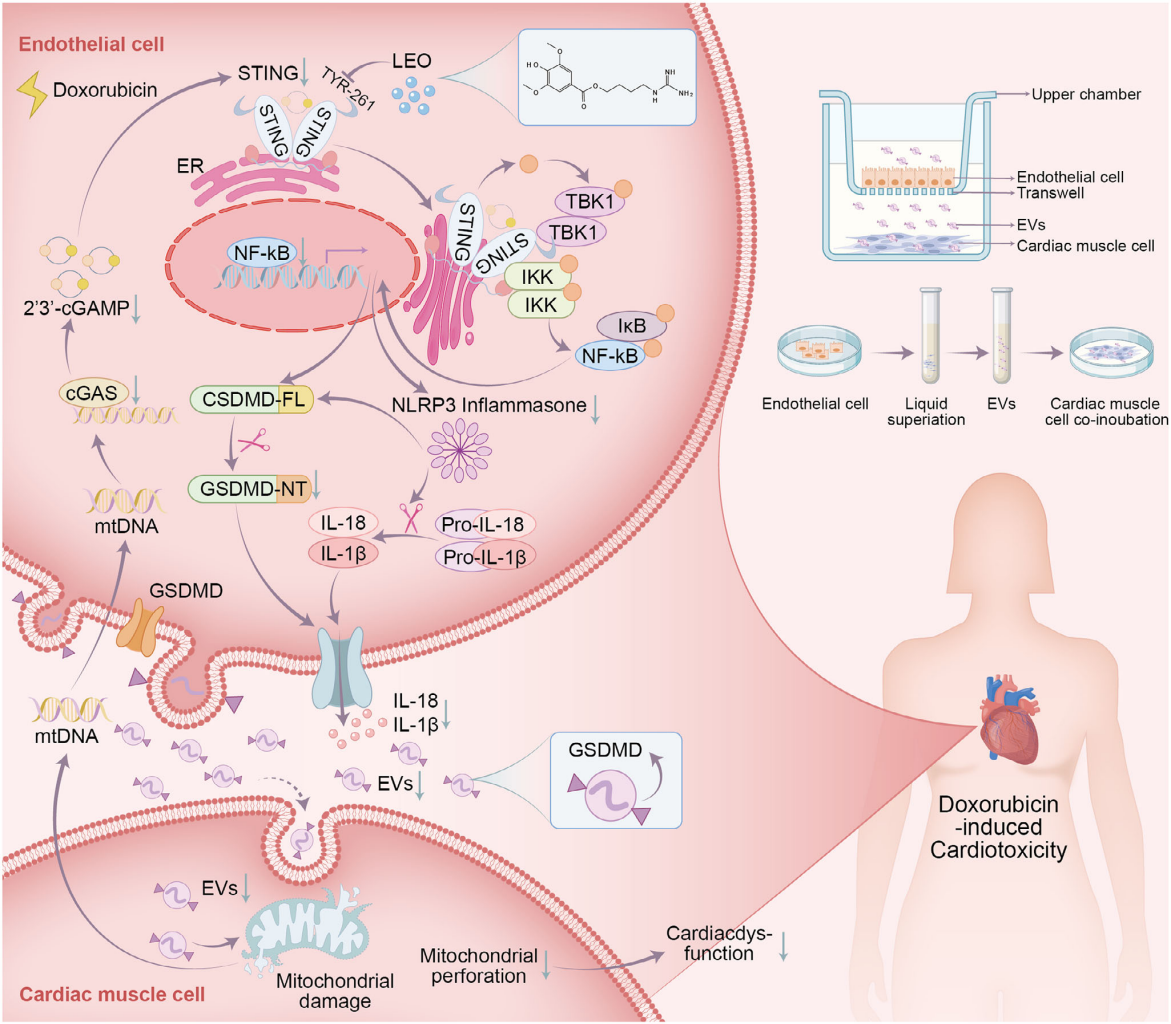
Schematic Diagram of the Mechanism of LEO in Treating DIC.

Our study elucidates a novel and self‐perpetuating pathogenic loop that critically exacerbates DIC, and identifies the natural compound LEO as a potent therapeutic agent that disrupts this loop by directly targeting the STING signaling pathway. As summarized in our proposed model (Figure 9), the mechanism unfolds as follows: DOX‐induced cardiac injury leads to mitochondrial damage in cardiomyocytes and the release of mtDNA into the circulation. This mtDNA is sensed by the cGAS‐STING axis in CVECs, triggering STING oligomerization and TBK1 recruitment [32]. The activated STING‐TBK1 complex then promotes NF‐κB‐mediated transcription of pro‐inflammatory genes and NLRP3 inflammasome assembly, culminating in GSDMD‐dependent pyroptosis. A critical consequence of this endothelial pyroptosis is the release of extracellular vesicles that act as pathogenic messengers, which are taken up by cardiomyocytes to induce profound mitochondrial dysfunction, thereby completing a vicious cycle that amplifies myocardial injury. The central finding of our research is that LEO intervenes precisely at the core of this cascade. We demonstrate that LEO directly binds to the TYR261 residue of STING, a highly conserved amino acid essential for its function [33, 34]. This binding sterically hinders STING oligomerization and disrupts the formation of the STING‐TBK1 heterodimer, effectively shutting down the downstream NF‐κB/NLRP3/GSDMD signaling network in CVECs. By inhibiting endothelial pyroptosis, LEO drastically reduces the production and release of the harmful EVs, thereby protecting cardiomyocytes from EVs ‐induced mitochondrial damage and metabolic deficits. The specificity of this mechanism is unequivocally confirmed by our experiments showing that the cardioprotective effects of LEO are completely abolished in STING‐overexpressing cells and, most importantly, are non‐additive in STING‐knockout mice, solidifying STING as the primary functional target of LEO in vivo.

Our study first establishes the potent therapeutic efficacy of LEO against DIC in vivo. The central clinical challenge of DIC is the progressive decline in cardiac function and adverse structural remodeling, for which specific treatments are lacking [35]. We demonstrated that LEO treatment dose‐dependently improved key echocardiographic parameters, enhanced survival, and mitigated pathological hallmarks of DIC, including cardiomyocyte atrophy, inflammation, and fibrosis. Crucially, LEO directly counteracted the profound mitochondrial damage induced by DOX, a cornerstone of its cardiotoxicity. These comprehensive in vivo pharmacodynamic data, spanning functional, histological, and ultrastructural analyses, firmly position LEO as a compelling candidate for mitigating the dose‐limiting toxicity of a widely used chemotherapeutic agent [36, 37].

Having established the therapeutic effect, we sought to delineate its molecular basis. The scientific question of which cell type and signaling pathway are primarily responsible for transducing the DOX injury signal remains pivotal. By integrating cardiac transcriptomics with isolated cell validation, we identified the cGAS/STING/NF‐κB/NLRP3 pathway as a primary target of LEO. The innovation here lies in our identification of CVECs as the critical cellular hub for this pathway's activation in DIC. Using multiple endothelial models (primary CMVEC and HUVEC), we showed that LEO inhibits this cascade at multiple levels, from gene expression to protein phosphorylation and inflammasome activation, ultimately suppressing GSDMD‐mediated pyroptosis. The definitive genetic evidence—that STING overexpression abolished LEO's benefits while STING knockdown phenocopied them—confirms the pathway's necessity and underscores the specificity of LEO's action on endothelial STING [38, 39].

The paramount question of how LEO engages the STING pathway was addressed through a rigorous structural biology and biophysics campaign. We moved beyond correlative association to demonstrate direct binding. Our innovative finding is that LEO specifically binds to the TYR261 residue of STING, a site crucial for both STING oligomerization and the subsequent formation of the STING‐TBK1 heterodimer. This binding mode is mechanistically distinct from known STING inhibitors such as H‐151 and C‐176, which covalently modify the Cys91 residue to block STING palmitoylation and trafficking. In contrast, LEO employs a non‐covalent, reversible interaction targeting TYR261, which may offer advantages in terms of target specificity and reduced off‐target effects. Furthermore, while H‐151 and C‐176 have been primarily investigated in short‐term inflammatory models with limited long‐term safety data, LEO—as the active alkaloid from the traditional herb *Leonurus japonicus*—demonstrated a favorable safety profile in our three‐month toxicity study, with no observed hepatotoxicity, nephrotoxicity, or cardiotoxicity. This was confirmed by an orthogonal suite of techniques: cellular CETSA showed target engagement in cells, SPR quantified binding affinity to the pure protein, and competitive pull‐down assays with both wild‐type and TYR261‐mutant STING established this single residue as essential. Kinetic analysis revealed a relatively slow dissociation rate (koff = 1.25 × 10^−^^2^ s^−^^1^, t_1_/_2_ ≈ 55 s), which allows LEO to maintain stable target engagement even at sub‑saturating concentrations and explains the potent cellular efficacy observed at 5–10 µM despite a moderate equilibrium KD (≈ 72 µM). The functional consequence is elegant: by occupying this strategic site, LEO acts as a molecular wedge, sterically hindering STING oligomerization and the subsequent formation of the productive STING/TBK1 heterodimer, as exquisitely detailed by our molecular dynamics simulations. This mechanism, from atomic‐level binding to functional complex disruption, represents a significant advancement in understanding how a natural product can precisely modulate a key innate immune signaling complex [40–42].

We next unraveled a novel intercellular communication axis that propagates injury from the endothelium to the myocardium. The critical scientific problem in DIC is understanding how localized endothelial damage leads to global cardiac dysfunction. We demonstrated that GSDMD‐mediated pyroptosis in CVECs results in the release of bioactive extracellular vesicles. Using a combination of TEM, NTA, and functional vesicle transfer experiments, we proved that these vesicles are not mere biomarkers but active vectors of injury, sufficient to induce mitochondrial ROS, dysfunction, and metabolic deficits in recipient cardiomyocytes. The most compelling evidence for this causal chain comes from our experiments with the GSDMD inhibitor DSF, which, by blocking pyroptosis, abolished the vesicle's toxicity. This establishes a direct link between endothelial STING/GSDMD activation and the profound mitochondrial pathology observed in cardiomyocytes, closing the pathogenic loop [43–45].

Our study outlines a comprehensive pathogenic pathway in DIC: DOX induces mtDNA release, which activates endothelial cGAS/STING (enhanced by LEO‐targeting TYR261), leading to NLRP3 inflammasome/GSDMD‐mediated pyroptosis, vesicle release, and subsequent cardiomyocyte mitochondrial dysfunction. The major innovation of this study is the integration of a direct STING‐targeting mechanism with a novel intercellular vesicle‐mediated communication pathway, both of which are disrupted by the natural product LEO. Our conclusions are robust due to the combined use of in vivo models, various cell types, and critical genetic manipulations, including knockdown, overexpression, and knockout. A limitation is that the relative contribution of other cardiac cell types to this loop warrants further exploration. Nevertheless, by identifying LEO as a specific STING oligomerization inhibitor and elucidating this endothelial‐centric vicious cycle, our findings not only provide a fundamental advance in the pathophysiology of DIC but also open a promising translational avenue for using LEO as a co‐therapy to enhance the safety of cancer chemotherapy.

## Author Contributions

**Li Chun**: funding acquisition, visualization, project administration, resources, writing – review and editing, writing – original draft. **He Jiale**: validation, software, data curation. **Yang Zhi**: validation, data curation, investigation. **Tao Meijiao**: methodology, supervision, investigation, formal analysis. **Wang Wei**: funding acquisition, visualization, project administration, resources, writing – original draft, writing – review and editing. **Zhao Jiangfeng**: conceptualization, investigation, methodology. **Xu Jianglin**: validation, visualization, formal analysis. **Wang Jun**: conceptualization, writing – original draft, writing – review and editing, validation. **Xu Xuegong**: writing – review and editing, writing – original draft, funding acquisition, visualization, project administration, resources. **Chen Xiaoyang**: methodology, project administration, writing – review and editing, writing – original draft. **Hei Xuanding**: validation, formal analysis.

## Ethics Statement

All experiments involving mice were performed in accordance with the ethical policies and procedures approved by the Animal Ethics Committee of Guangzhou University of Chinese Medicine (approval No. 20240926009).

## Conflicts of Interest

The authors declare no conflicts of interest.

## Supporting information




**Supporting File**: advs75912‐sup‐0001‐SuppMat.pdf.

## Data Availability

The data that support the findings of this study are available from the corresponding author upon reasonable request.
