# Supplementary material for: Leonurine Ameliorates Doxorubicin‐Induced Cardiotoxicity via STING/NF‐κB/NLRP3 Inflammasome Signaling Pathway
Source: Adv Sci (Weinh). 2026 Jun 12:e75912. Online ahead of print. doi: 10.1002/advs.75912 (PMC13336502; doi:10.1002/advs.75912)
Supplement: Supplementary file 1 — Supporting File: advs75912‐sup‐0001‐SuppMat.pdf. [file ADVS-9999-e75912-s001.pdf]

Supplementary materials

1 Primer sequences

Table S1. The Primer sequences information in this study

Primer Name	Primer Sequence（5`-3`）	Amplicon Size
Mouse cGAS	Forward: GAGGCGCGGAAAGTCGTAA<br>Reverse: TTGTCCGGTTCCTTCCTGGA	98
Mouse STING	Forward: GGCTGGCCTGGTCATACTAC<br>Reverse: GCACCACTGAGCATGTTGTT	106
Mouse TBK1	Forward: ACTGGTGATCTCTATGCTGTCA<br>Reverse: TTCTGGAAGTCCATACGCATTG	237
Mouse IKK	Forward: ACAGCCAGGAGATGGTACG<br>Reverse: CAGGGTGACTGAGTCGAGAC	297
Mouse NF-κB	Forward: ATGGCAGACGATGATCCCTAC<br>Reverse: TGTTGACAGTGGTATTTCTGGTG	111
Mouse NLRP3	Forward: ATTACCCGCCCGAGAAAGG<br>Reverse: TCGCAGCAAAGATCCACACAG	141
Mouse ASC	Forward: CTTGTCAGGGGATGAACTCAAAA<br>Reverse: GCCATACGACTCCAGATAGTAGC	154
Mouse CASP1	Forward: ACAAGGCACGGGACCTATG<br>Reverse: TCCCAGTCAGTCCTGGAAATG	237
Mouse GSDMD	Forward: CCATCGGCCTTTGAGAAAGTG<br>Reverse: ACACATGAATAACGGGGTTTCC	170
Mouse IL18	Forward: GACTCTTGCGTCAACTTCAAGG<br>Reverse: CAGGCTGTCTTTTGTCAACGA	169
Mouse IL1β	Forward: GCAACTGTTCCTGAACTCAACT<br>Reverse: ATCTTTTGGGGTCCGTCAACT	89
Mouse TNFα	Forward: CCCTCACACTCAGATCATCTTCT<br>Reverse: GCTACGACGTGGGCTACAG	61
Mouse	Forward: GTGCGGTGTCCAACACAGAT	329

ANP	Reverse: TCCAATCCTGTCAATCCTACCC	
Mouse	Forward: GAGGTCACTCCTATCCTCTGG	100
BNP	Reverse: GCCATTTCCTCCGACTTTTCTC	
Mouse	Forward: ACTGTCAACACTAAGAGGGTCA	114
β-MHC	Reverse: TTGGATGATTTGATCTTCCAGGG	
Mouse	Forward: GTCCCAGACATCAGGGAGTAA	102
ACTA2	Reverse: TCGGATACTTCAGCGTCAGGA	
Mouse	Forward: CAGCCTTCCACTAAGGATTTGC	120
FN1	Reverse: GCTCTAAACCCCTTCTCTCTGAG	
Mouse	Forward: CTGGACAGCCAGACACTAAAG	145
MMP9	Reverse: CTCGCGGCAAGTCTTCAGAG	
Mouse	Forward: ACTGACATCCTCGCTTCTGAA	124
TGF-β	Reverse: ATACGTTTCCTCTCAAAACCCC	
Mouse	Forward: TTCTTCCGCCAGTCGGTAG	141
PINK	Reverse: CTGCTTCTCCTCGATCAGCC	
Mouse	Forward: TCTTCCAGTGTAACCACCGTC	115
PARKIN	Reverse: GCAGGGAGTAGCCAAGTT	
Mouse	Forward: GGCTCACATCGTTGATACTTGG	70
DRP1	Reverse: GCCACATTCAGGCTGTACTGTA	
Mouse	Forward: CGTGATTAGCGATGATGAACCA	89
Mt-CO1	Reverse: TCCAAATCCTCGGCATAATGA	
Mouse	Forward: CAGGAAGACACTGCACTTTGA	78
MT-CO2	Reverse: TTCAGGAAGAGCCACACTTCT	
Mouse	Forward: TCCTTCGGCCTTCTTCTTGTT	35
MT-ATP6	Reverse: AGGATGCTCGCCTTTGATTTT	
Mouse	Forward: AGGTCGGTGTGAACGGATTTG	123
GAPDH	Reverse: TGTAGACCATGTAGTTGAGGTCA	
Human	Forward: CACGAAGCCAAGACCTCCG	135
cGAS	Reverse: GTCGCACTTCAGTCTGAGCA	

Human STING	Forward: CCAGAGCACACTCTCCGGTA<br>Reverse: CGCATTTGGGAGGGAGTAGTA	145
Human TBK1	Forward: TGGGTGGAATGAATCATCTACGA<br>Reverse: GCTGCACCAAAATCTGTGAGT	67
Human NF-κB	Forward: AACAGAGAGGATTTCGTTTCCG<br>Reverse: TTTGACCTGAGGGTAAGACTTCT	89
Human NLRP3	Forward: GATCTTCGCTGCGATCAACAG<br>Reverse: CGTGCATTATCTGAACCCCAC	83
Human GSDMD	Forward: GTGTGTCAACCTGTCTATCAAGG<br>Reverse: CATGGCATCGTAGAAGTGGAAG	23
Human CASP1	Forward: TTTCCGCAAGGTTCGATTTTCA<br>Reverse: GGCATCTGCGCTCTACCATC	45
Human GAPDH	Forward: GGAGCGAGATCCCTCCAAAAT<br>Reverse: GGCTGTTGTCATACTTCTCATGG	101

2. LEO Improves Cardiac Fibrosis and Myocardial Injury in DIC Mice

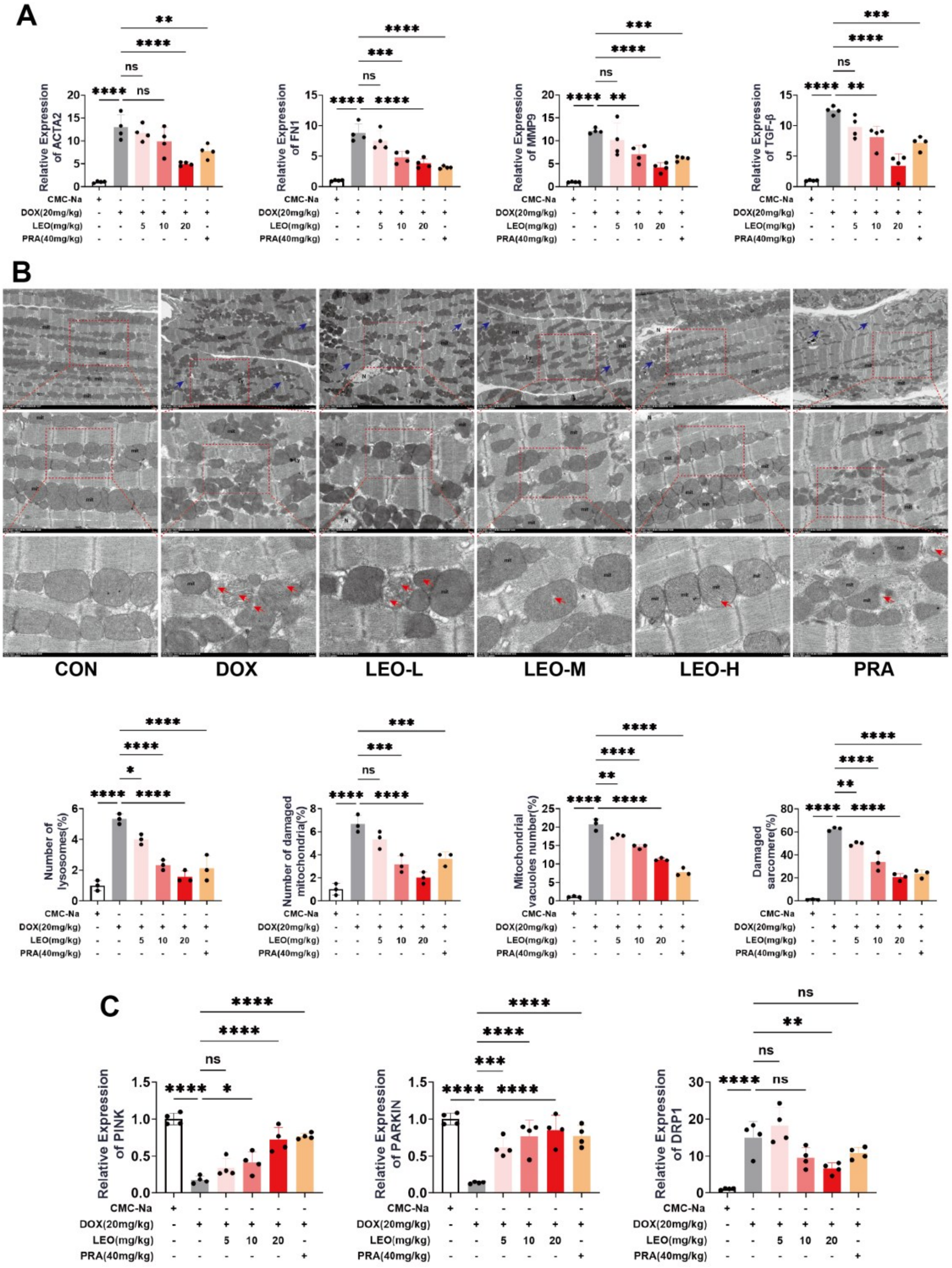

Figure S1. LEO attenuates cardiac fibrosis and myocardial injury in DIC mice. (A) Expression levels of ACTA2, FN1, MMP9, and TGF-β in mouse hearts. (B) Transmission electron microscopy (TEM) and statistical analysis of mouse hearts. Red

arrow: Mitochondrial vacuole; Blue arrow: Myofilament rupture; Ly: Lysosome; mit: Mitochondrion.(C) Relative mRNA expression levels of PINK, PARKIN, and DRP1 in mouse hearts. $^{*}P < 0.05$, $^{**}P < 0.01$, $^{***}P < 0.001$, $^{****}P < 0.01$ and ns: Not significant.

3 LEO significantly improved the survival prognosis of DIC mice

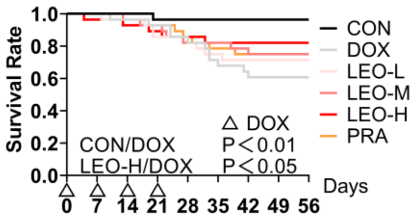

Figure S2. LEO significantly improved the survival prognosis of DIC mice.

4 Transcriptomic analysis of mouse heart

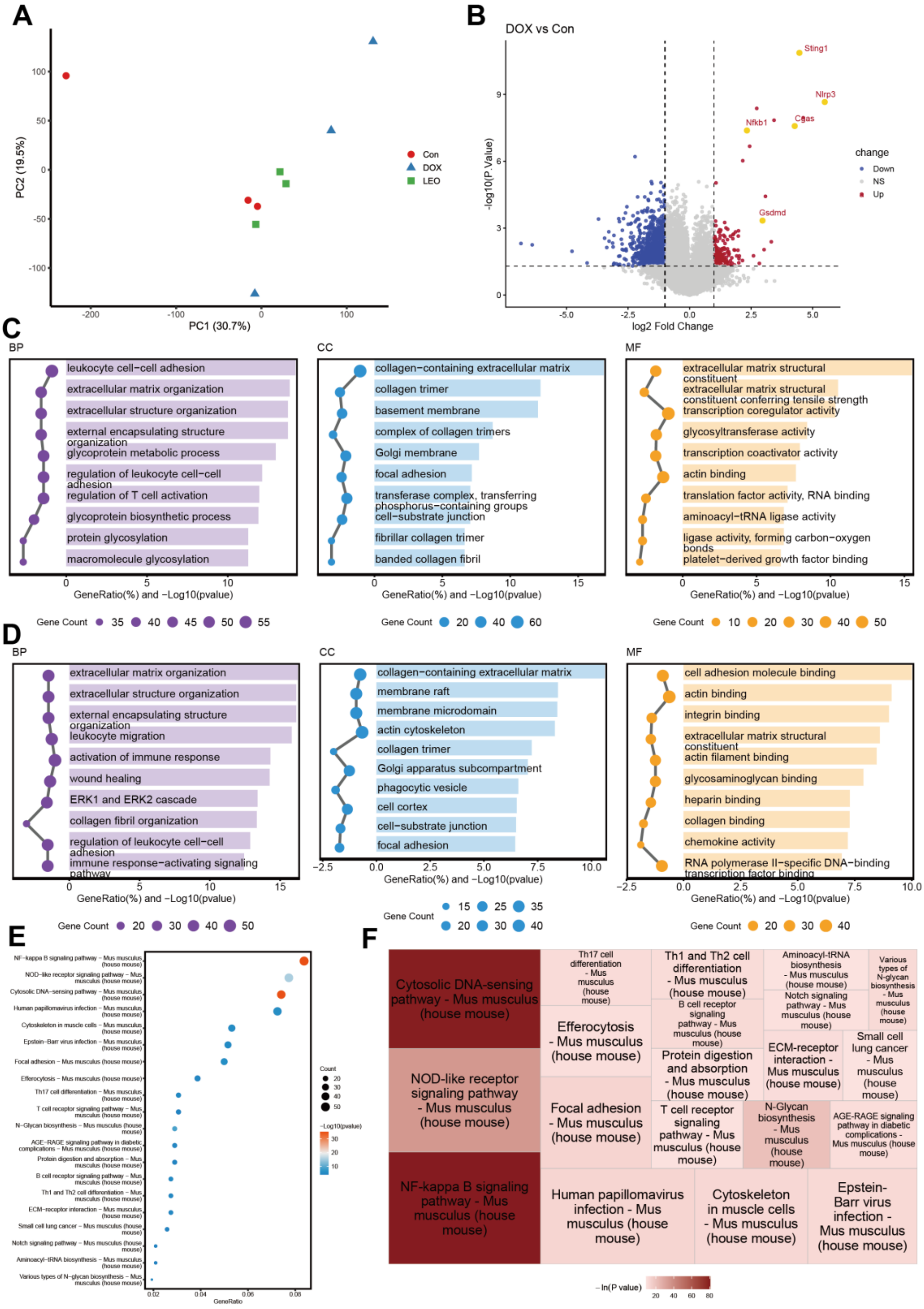

Figure S3. Transcriptomic analysis of hearts from DIC mice. (A) Principal component analysis (PCA) of transcriptomic differences among the three groups. (B) Volcano plot of differentially expressed genes between the DOX group and the CON group. (C) Gene

Ontology (GO) enrichment analysis of differentially expressed genes between the DOX group and the CON group. (D) GO enrichment analysis of differentially expressed genes between the LEO group and the DOX group. (E) Bubble plot of Kyoto Encyclopedia of Genes and Genomes (KEGG) enrichment analysis for differentially expressed genes between the DOX group and the CON group. (F) Heatmap of KEGG enrichment analysis for differentially expressed genes between the DOX group and the CON group.

5 Results of scanning electron microscopy of mouse cardiac blood vessels and levels of free mitochondrial DNA (mtDNA) in the heart

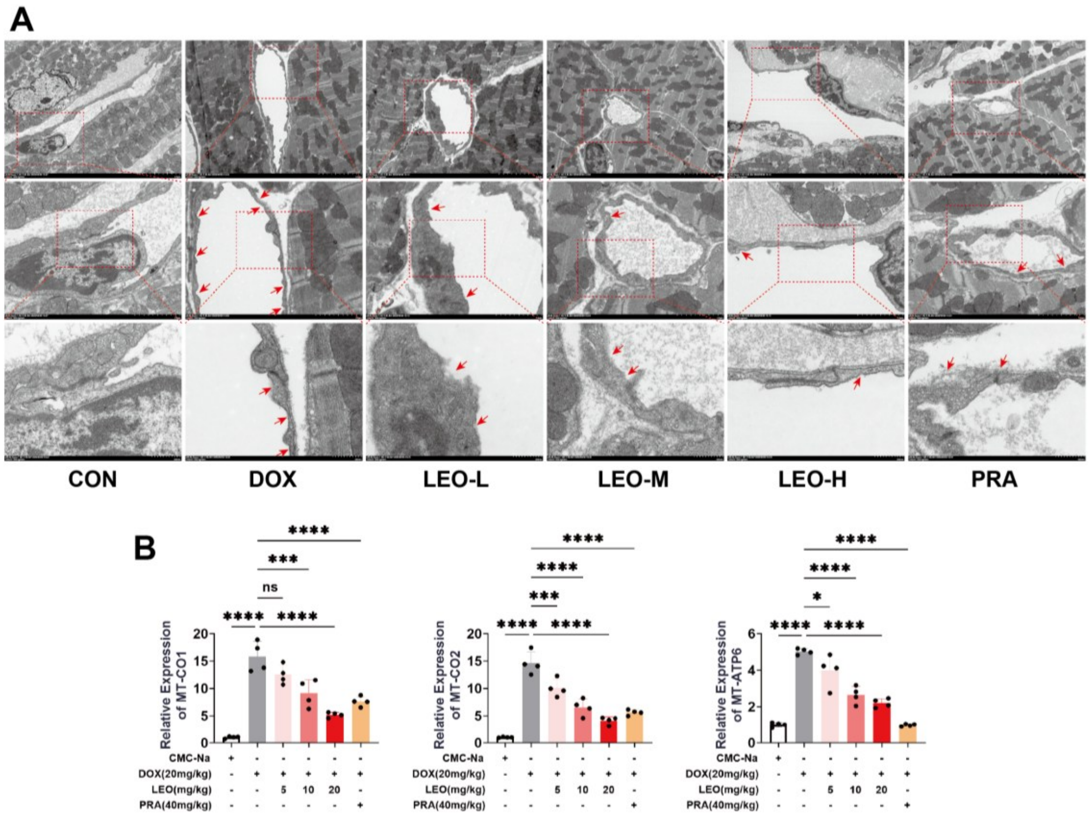

Figure S4. Scanning electron microscopy results of mouse cardiac blood vessels and levels of cardiac mitochondrial DNA (mtDNA). (A) Scanning electron microscopy images of mouse cardiac blood vessels and evaluation of endothelial tight junctions. (B) Levels of free mtDNA in mouse hearts. $^{*}P < 0.05$, $^{**}P < 0.01$, $^{***}P < 0.001$, $^{****}P < 0.01$ and ns: Not significant.

6 Immunofluorescence of STING/TBK1/NF-κB/NLRP3/CASP1 signaling in CMVECs

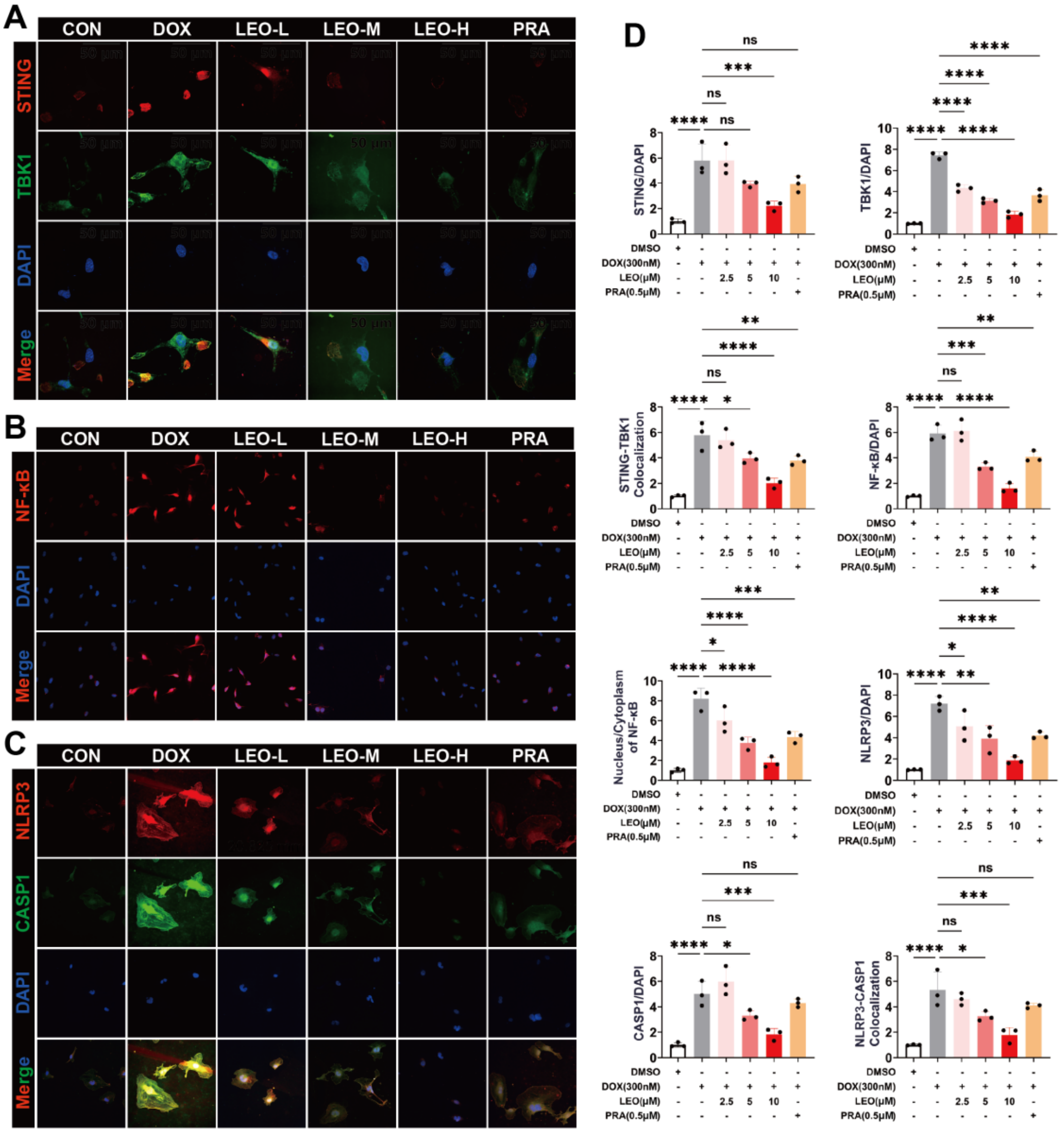

Figure S5. Immunofluorescence of STING/TBK1/NF-κB/NLRP3/CASP1 signaling in CMVECs. (A) Immunofluorescence of STING/TBK1 in CMVECs. (B) Immunofluorescence of NF-κB in CMVECs. (C) Immunofluorescence of NLRP3/CASP1 in CMVECs. (D) Statistical analysis of immunofluorescence for STING/TBK1/NF-κB/NLRP3/CASP1 signaling in CMVECs. $^{*}P < 0.05$, $^{**}P < 0.01$, $^{***}P < 0.001$, $^{****}P < 0.01$ and ns: Not significant.

7 The impact of STING overexpression on the ameliorative effects of LEO on DOX-induced CMVEC injury

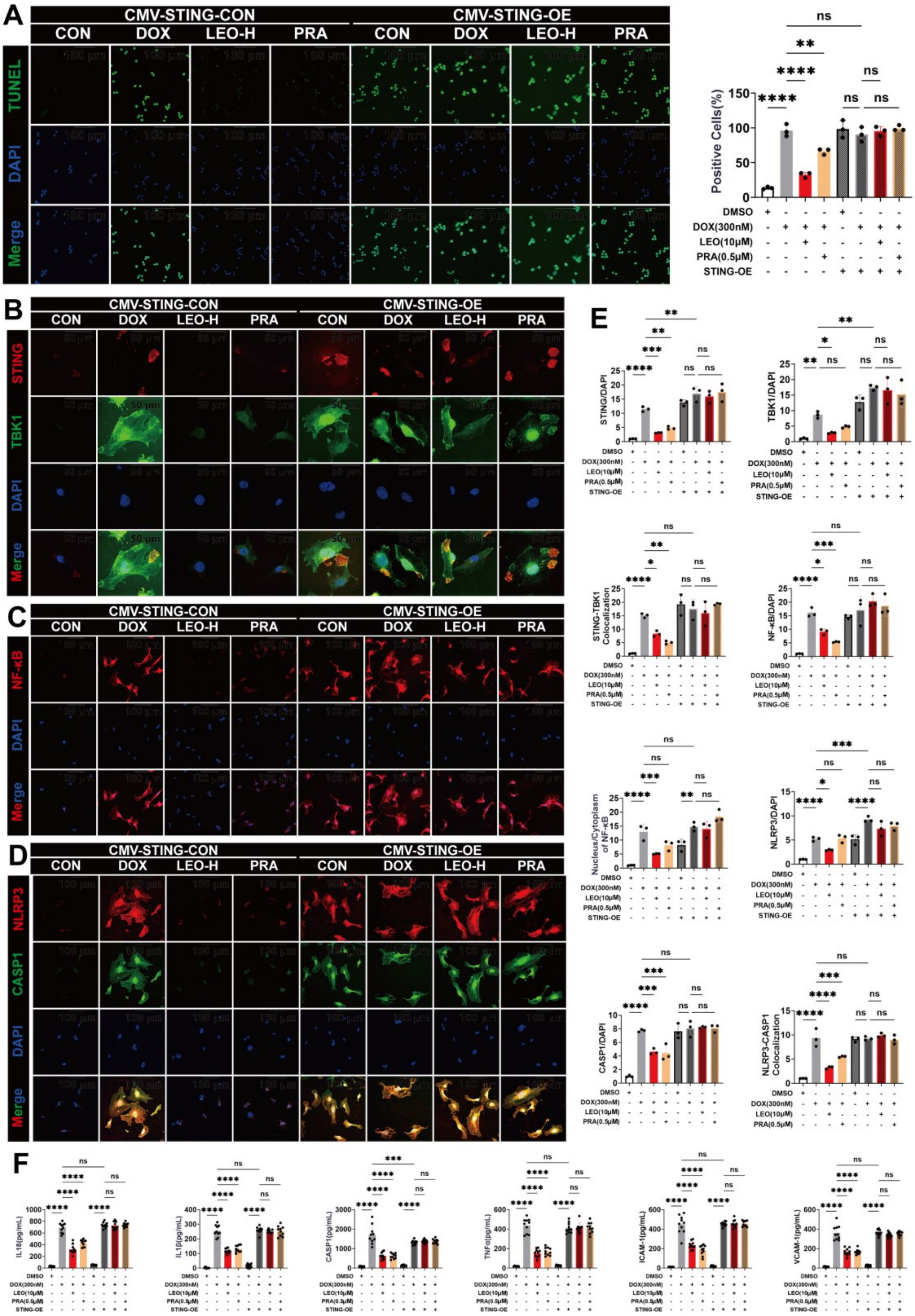

Figure S6. The impact of STING overexpression on LEO's amelioration of DOX-induced CMVEC injury. (A) Tunel staining of CMVECs under STING overexpression.

(B) Immunofluorescence of STING/TBK1 in CMVECs under STING overexpression. (C) Immunofluorescence of NF-κB in CMVECs under STING overexpression. (D) Immunofluorescence of NLRP3/CASP1 in CMVECs under STING overexpression. (E) Statistical analysis of immunofluorescence for STING/TBK1/NF-κB/NLRP3/CASP1 signaling in CMVECs under STING overexpression. (F) Levels of IL18, IL1β, CASP1, TNFα, ICAM-1, and VCAM-1 in the supernatant of CMVEC culture medium under STING overexpression. $^{*}P < 0.05$, $^{**}P < 0.01$, $^{***}P < 0.001$, $^{****}P < 0.01$ and ns: Not significant.

8 The impact of STING knockdown on LEO's amelioration of DOX-induced CMVEC injury

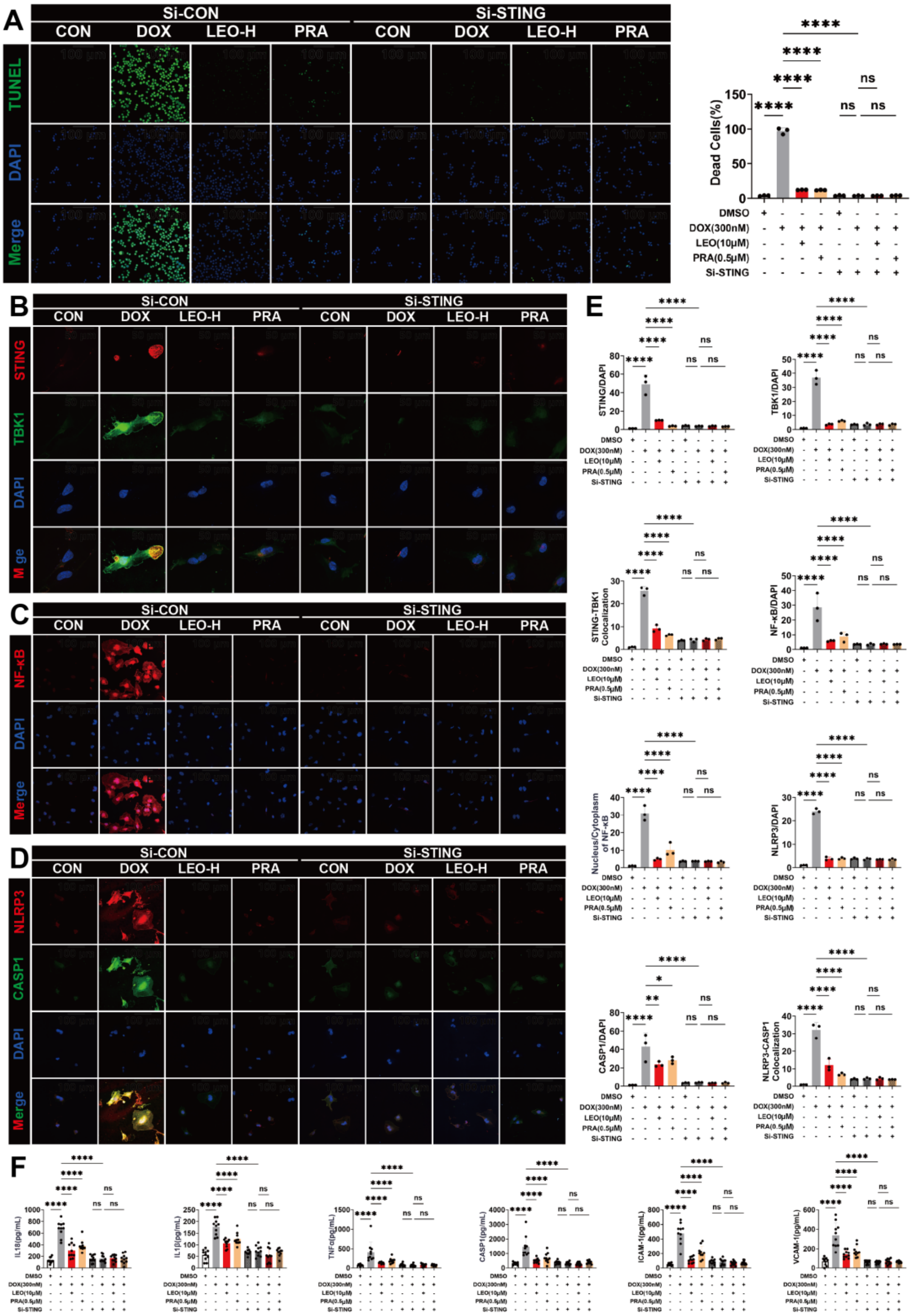

Figure S7. The impact of STING knockdown on LEO's amelioration of DOX-induced CMVEC injury. (A) Tunel staining of CMVECs under STING knockdown. (B)

Immunofluorescence of STING/TBK1 in CMVECs under STING knockdown. (C) Immunofluorescence of NF-κB in CMVECs under STING knockdown. (D) Immunofluorescence of NLRP3/CASP1 in CMVECs under STING knockdown. (E) Statistical analysis of immunofluorescence for STING/TBK1/NF-κB/NLRP3/CASP1 signaling in CMVECs under STING knockdown. (F) Levels of IL18, IL1β, CASP1, TNFα, ICAM-1, and VCAM-1 in the supernatant of CMVEC culture medium under STING knockdown. $^{*}P < 0.05$, $^{**}P < 0.01$, $^{***}P < 0.001$, $^{****}P < 0.01$ and ns: Not significant.

9 LEO ameliorates DOX-mediated HUVEC injury through the cGAS/STING pathway

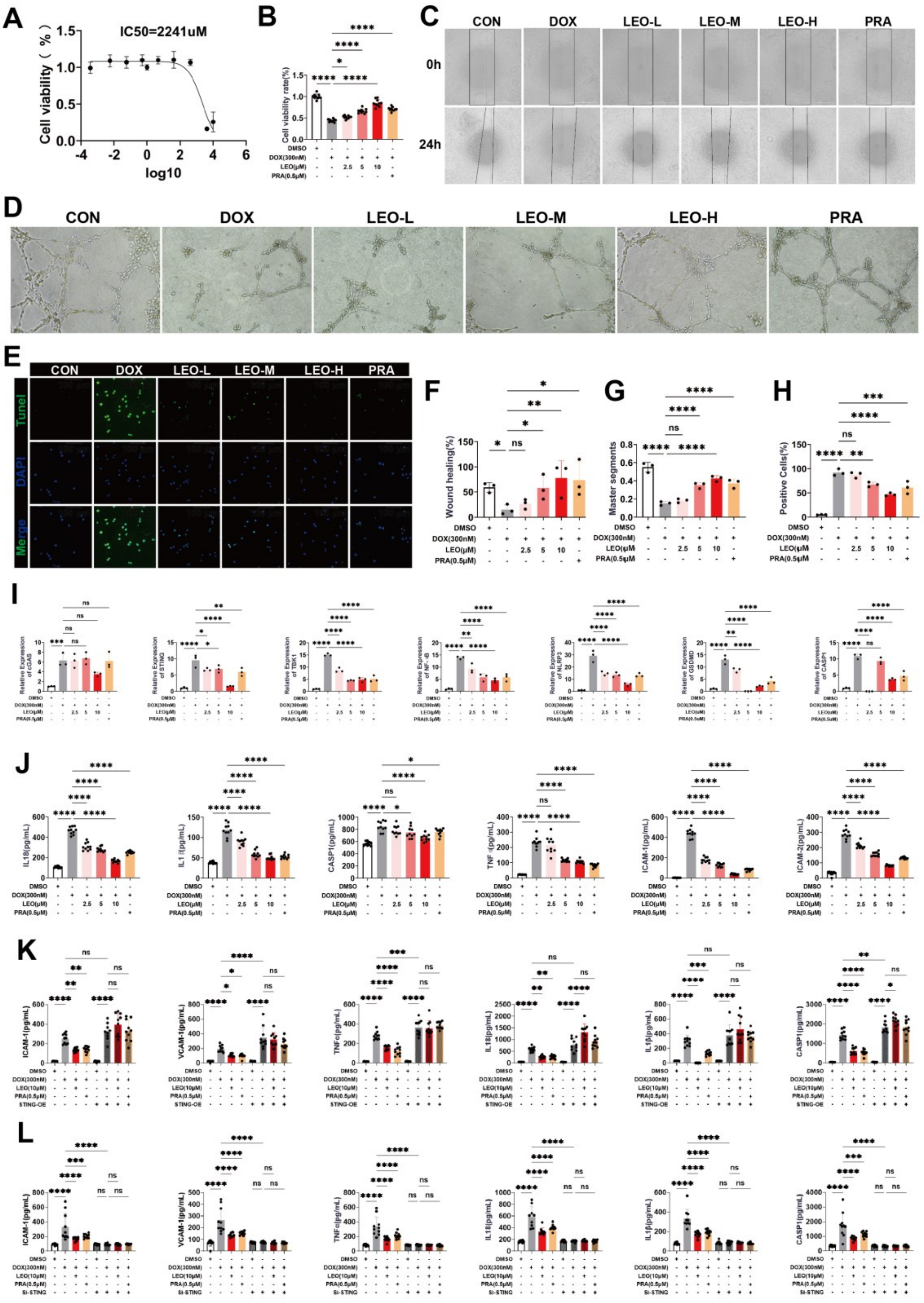

Figure S8. LEO ameliorates DOX-mediated HUVEC injury through the cGAS/STING pathway. (A) IC50 of LEO on HUVECs. (B) CCK8 assays. (C) Cell wound-healing

assay. (D) Tube formation assay. (E) TUNEL staining. (F) Statistical results of the cell wound-healing assay. (G) Statistical results of the tube formation assay. (H) Statistics on the positive cell rate of TUNEL staining. (I) Relative mRNA expression levels of cGAS, STING, TBK1, IKKα, NF-κB, ASC, NLRP3, GSDMD, and CASP1. (J) Levels of IL18, IL1β, TNFα, ICAM-1, ICAM-2, and CASP1 in the supernatant of HUVEC culture medium. (K) Effects of STING overexpression on the levels of IL18, IL1β, TNFα, ICAM-1, ICAM-2, and CASP1 in the supernatant of HUVEC culture medium. (L) Effects of STING knockdown on the levels of IL18, IL1β, TNFα, ICAM-1, ICAM-2, and CASP1 in the supernatant of HUVEC culture medium. $^{*}P < 0.05$, $^{**}P < 0.01$, $^{***}P < 0.001$, $^{****}P < 0.01$ and ns: Not significant.

10 Kinetic analysis of LEO and human STING protein mutant analysi

A

System	ΔE_{vdW}	ΔE_{elec}	ΔG_{GB}	ΔG_{SA}	ΔG_{bind}
**E260A**	-22.37±3.01	-21.41±8.86	31.10±7.42	-3.15±0.52	-15.83±1.11
**L212A**	-19.57±1.70	-43.91±8.12	49.59±7.25	-1.18±0.29	-15.07±1.44
**Q266A**	-23.60±3.38	-50.06±23.05	61.75±20.88	-3.58±0.27	-15.50±1.13
**S243A**	-25.61±3.62	-58.60±10.46	73.48±9.12	-3.50±0.60	-14.23±0.88
**T263A**	-26.56±3.38	-53.06±12.38	66.21±11.92	-3.89±0.17	-17.30±1.13
**Y245A**	-27.99±2.16	-50.08±8.41	64.85±7.53	-4.06±0.24	-17.28±1.10
**Y261A**	-20.01±3.75	-56.66±12.92	66.92±10.38	-3.30±0.65	-13.05±1.49

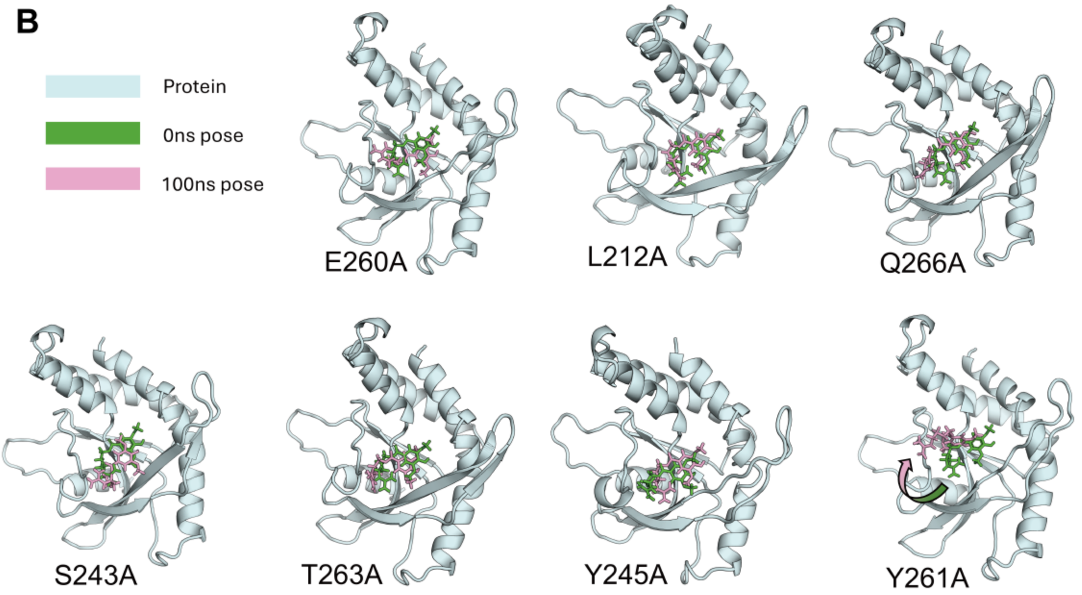

Figure S9. Kinetic analysis of LEO and human STING protein mutants. (A) Molecular dynamics MM - GBSA analysis of LEO and human STING protein. (B) Conformational changes in the molecular dynamics of LEO and human STING protein (100 ns).

11 The impact of LEO on 2’3’-cGAMP-mediated cell damage of CMVEC under STING overexpression conditions

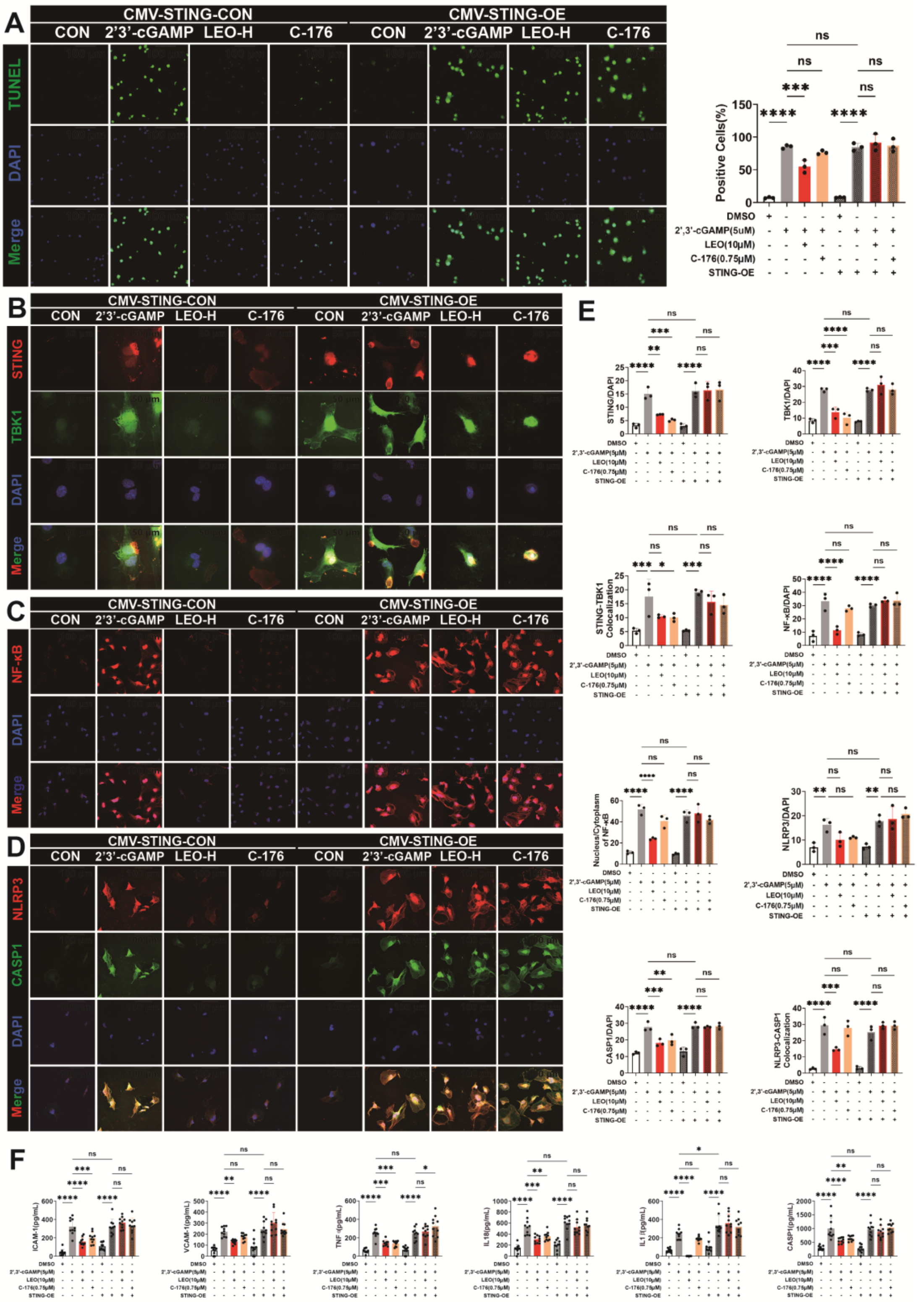

Figure S10. Effects of LEO on 2’3’-cGAMP-mediated cell damage of CMVEC under STING overexpression. (A) Tunel staining of CMVECs under STING overexpression.

(B) Immunofluorescence of STING/TBK1 in CMVECs under STING overexpression. (C) Immunofluorescence of NF-κB in CMVECs under STING overexpression. (D) Immunofluorescence of NLRP3/CASP1 in CMVECs under STING overexpression. (E) Statistical analysis of immunofluorescence signals for STING/TBK1/NF-κB/NLRP3/CASP1 in CMVECs under STING overexpression. (F) Levels of IL18, IL1β, CASP1, TNFα, ICAM-1, and VCAM-1 in the culture supernatant of CMVECs under STING overexpression. $^{*}P < 0.05$, $^{**}P < 0.01$, $^{***}P < 0.001$, $^{****}P < 0.01$ and ns: Not significant.

12 Effects of LEO on 2’3’-cGAMP-mediated cell damage of CMVEC under STING knockdown

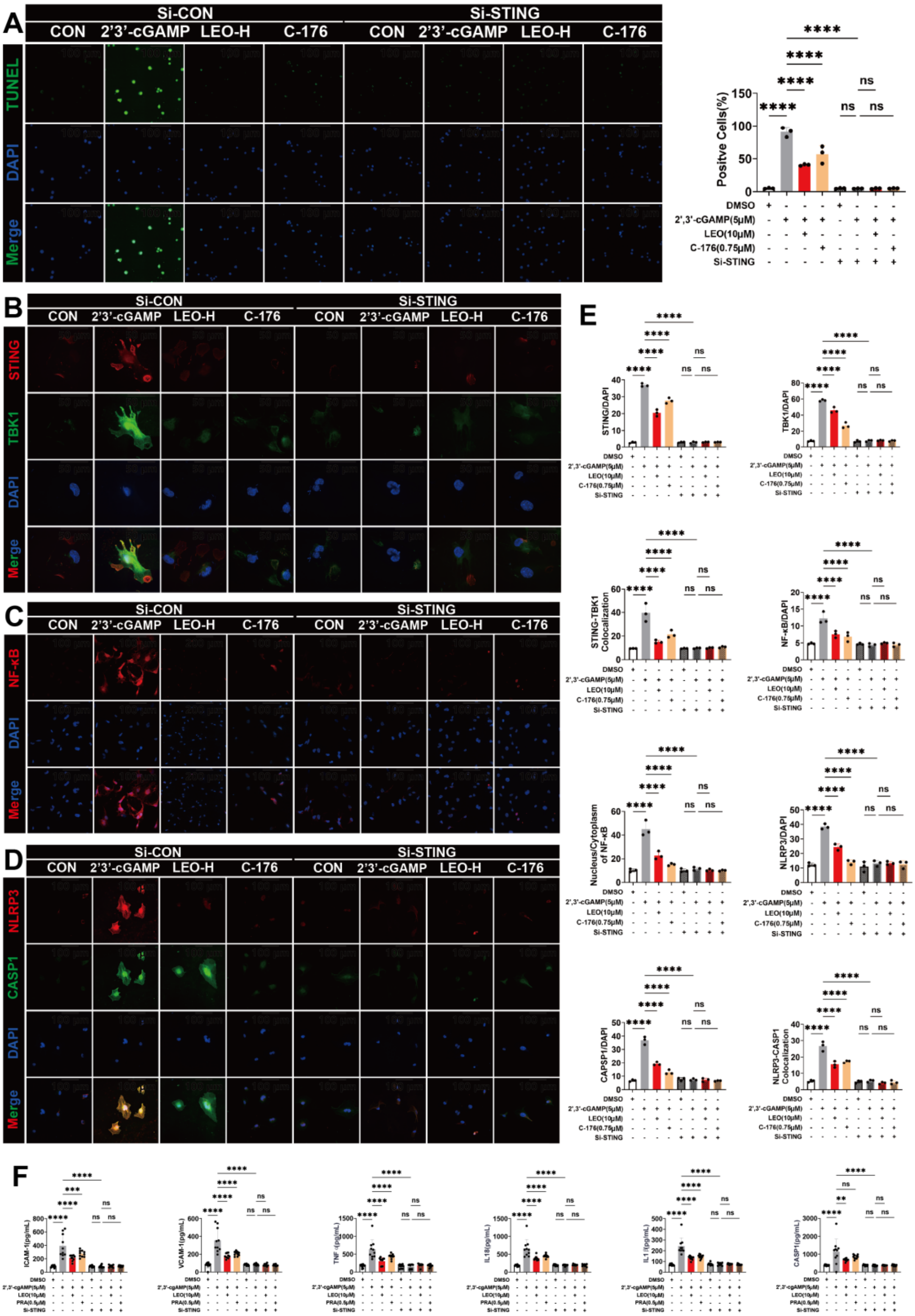

Figure S11. Effects of LEO on 2’3’-cGAMP-mediated cell damage of CMVEC under STING knockdown. (A) Tunel staining of CMVECs under STING overexpression

(Note: This should likely be "knockdown" to match the title; if so, correct to "under STING knockdown"). (B) Immunofluorescence of STING/TBK1 in CMVECs under STING knockdown (corrected from "overexpression"). (C) Immunofluorescence of NF-κB in CMVECs under STING knockdown (corrected from "overexpression"). (D) Immunofluorescence of NLRP3/CASP1 in CMVECs under STING knockdown (corrected from "overexpression"). (E) Statistical analysis of immunofluorescence signals for STING/TBK1/NF-κB/NLRP3/CASP1 in CMVECs under STING knockdown (corrected from "overexpression"). (F) Levels of IL18, IL1β, CASP1, TNFα, ICAM-1, and VCAM-1 in the culture supernatant of CMVECs under STING knockdown (corrected from "overexpression"). $^{*}P < 0.05$, $^{**}P < 0.01$, $^{***}P < 0.001$, $^{****}P < 0.01$ and ns: Not significant.

13 LEO ameliorates 2’3’-cGAMP-mediated HUVEC injury via the cGAS/STING pathway

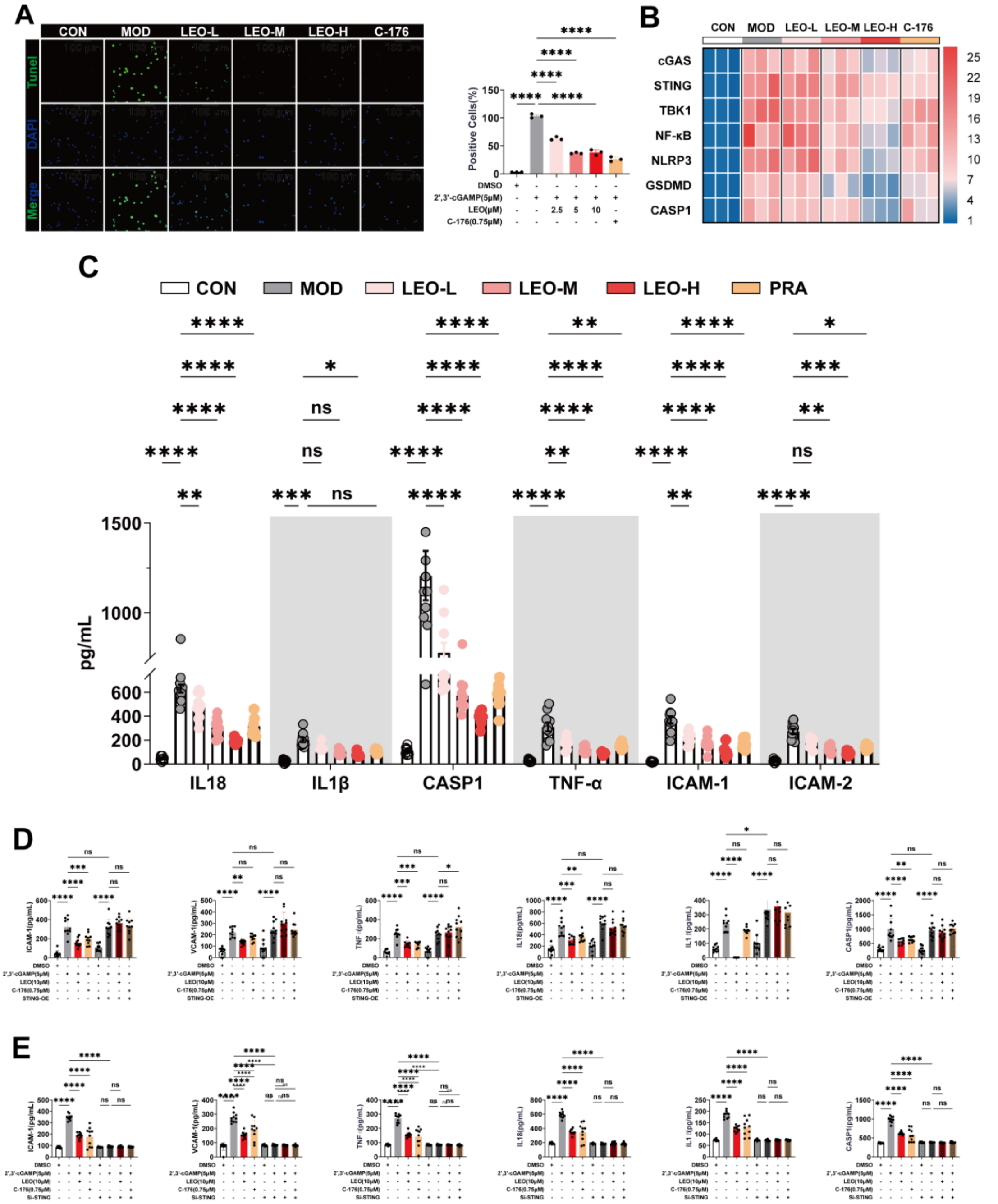

Figure S12. LEO ameliorates 2’3’-cGAMP-mediated HUVEC injury via the cGAS/STING pathway. (A) Tunel staining and statistical analysis. (B) Relative mRNA expression levels of cGAS, STING, TBK1, IKKα, NF-κB, ASC, NLRP3, GSDMD, and CASP1. (C) Levels of IL18, IL1β, TNFα, ICAM-1, ICAM-2, and CASP1 in the culture supernatant of HUVECs. (D) Effects of STING overexpression on the levels of IL18, IL1β, TNFα, ICAM-1, ICAM-2, and CASP1 in the culture supernatant of HUVECs. (E)

Effects of STING knockdown on the levels of IL18, IL1β, TNFα, ICAM-1, ICAM-2, and CASP1 in the culture supernatant of HUVECs. $^{*}P < 0.05$, $^{**}P < 0.01$, $^{***}P < 0.001$, $^{****}P < 0.01$ and ns: Not significant.

14 Effects of Vesicle Transplantation on Energy Metabolism in Cardiomyocytes

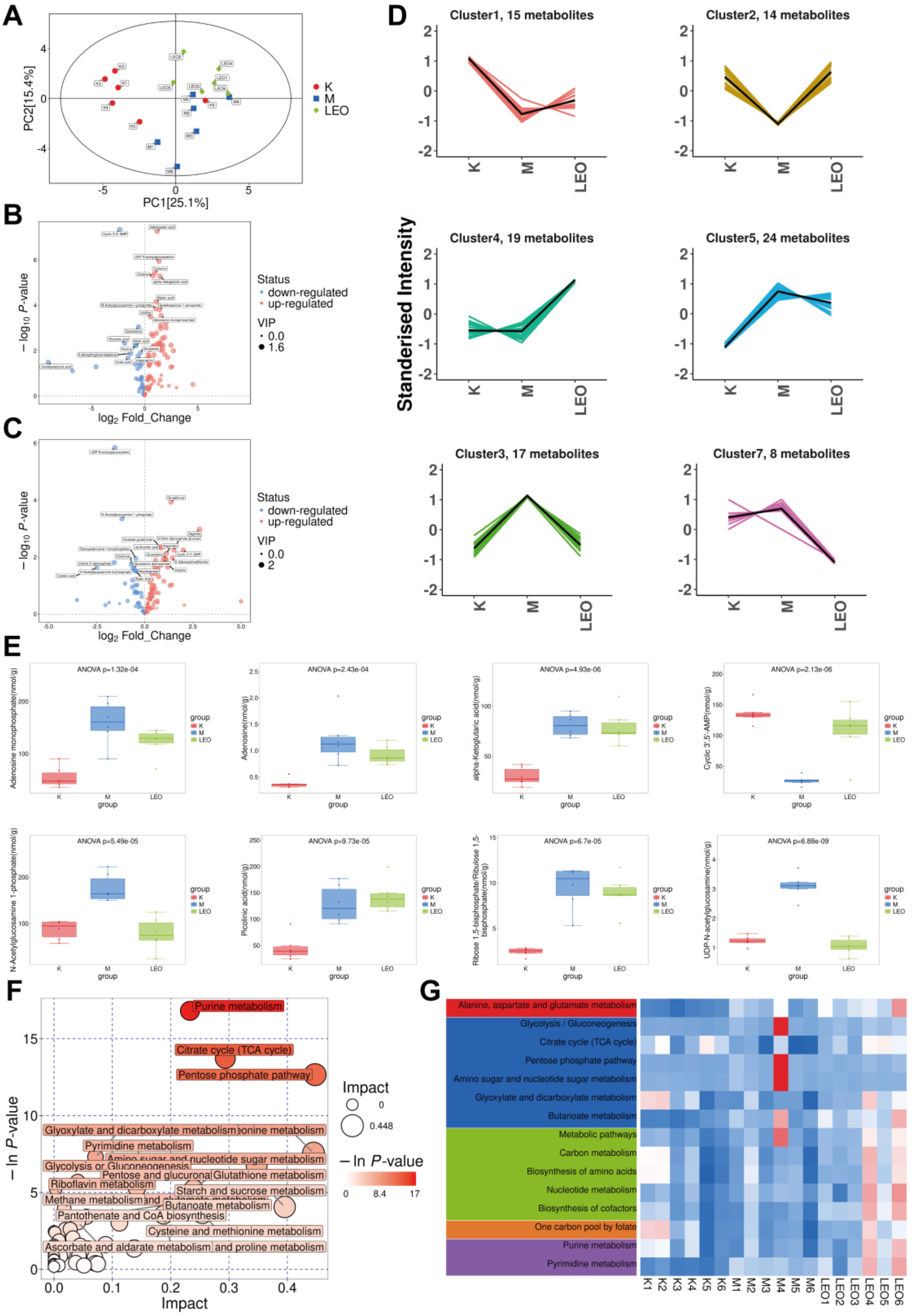

Figure S13. Effects of Vesicle Transplantation on Energy Metabolism in Cardiomyocytes. (A) PCA analysis of differential metabolites among the three groups. (B) Volcano plot of differential metabolites between the CON group and DOX group.

(C) Volcano plot of differential metabolites between the LEO group and DOX group. (D) Cluster analysis of differential metabolite levels among the three groups. (E) Box plot analysis of differential metabolite levels among the three groups. (F) Bubble plot of KEGG enrichment analysis for differential metabolites between the LEO group and DOX group. (G) Heatmap of KEGG enrichment analysis for differential metabolites between the DOX group and CON group.

15 Effects of STING Overexpression or Disulfiram on Vesicle Transplantation in Cardiomyocytes

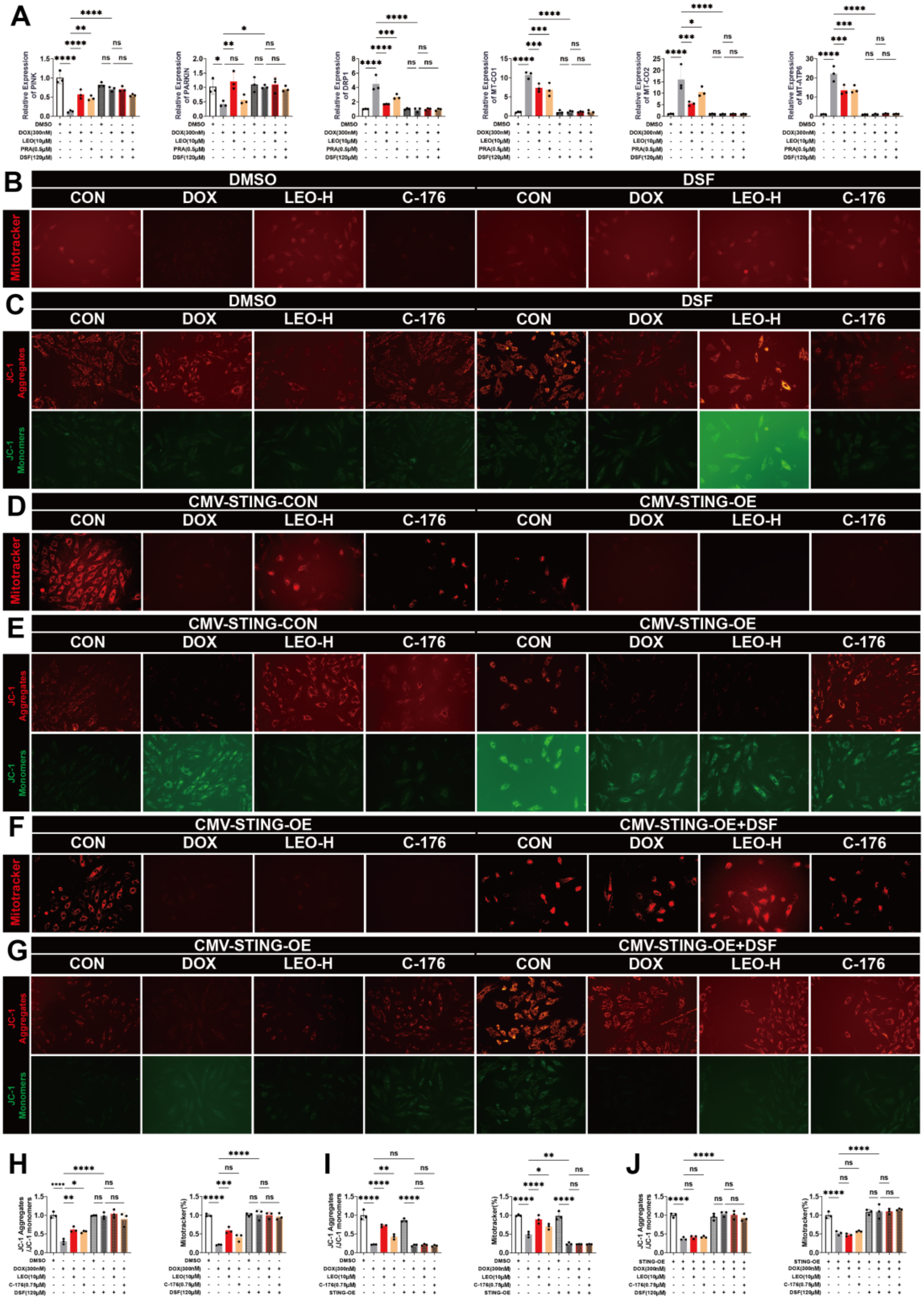

Figure S14. Effects of STING Overexpression or Disulfiram on Vesicle Transplantation in Cardiomyocytes. (A) Impact of disulfiram on the expression levels of PINK,

PARKIN, DRP1, MT-CO1, MT-CO2, and MT-ATP6 in cardiomyocytes subjected to vesicle transplantation. (B-J) Effects of STING overexpression or/and disulfiram on JC-1 and mitotracker staining in cardiomyocytes subjected to vesicle transplantation. $^{*}P < 0.05$, $^{**}P < 0.01$, $^{***}P < 0.001$, $^{****}P < 0.01$ and ns: Not significant.

16 LEO improves cardiac inflammation, fibrosis, ventricular remodeling and serum cardiac enzymes in DIC mice

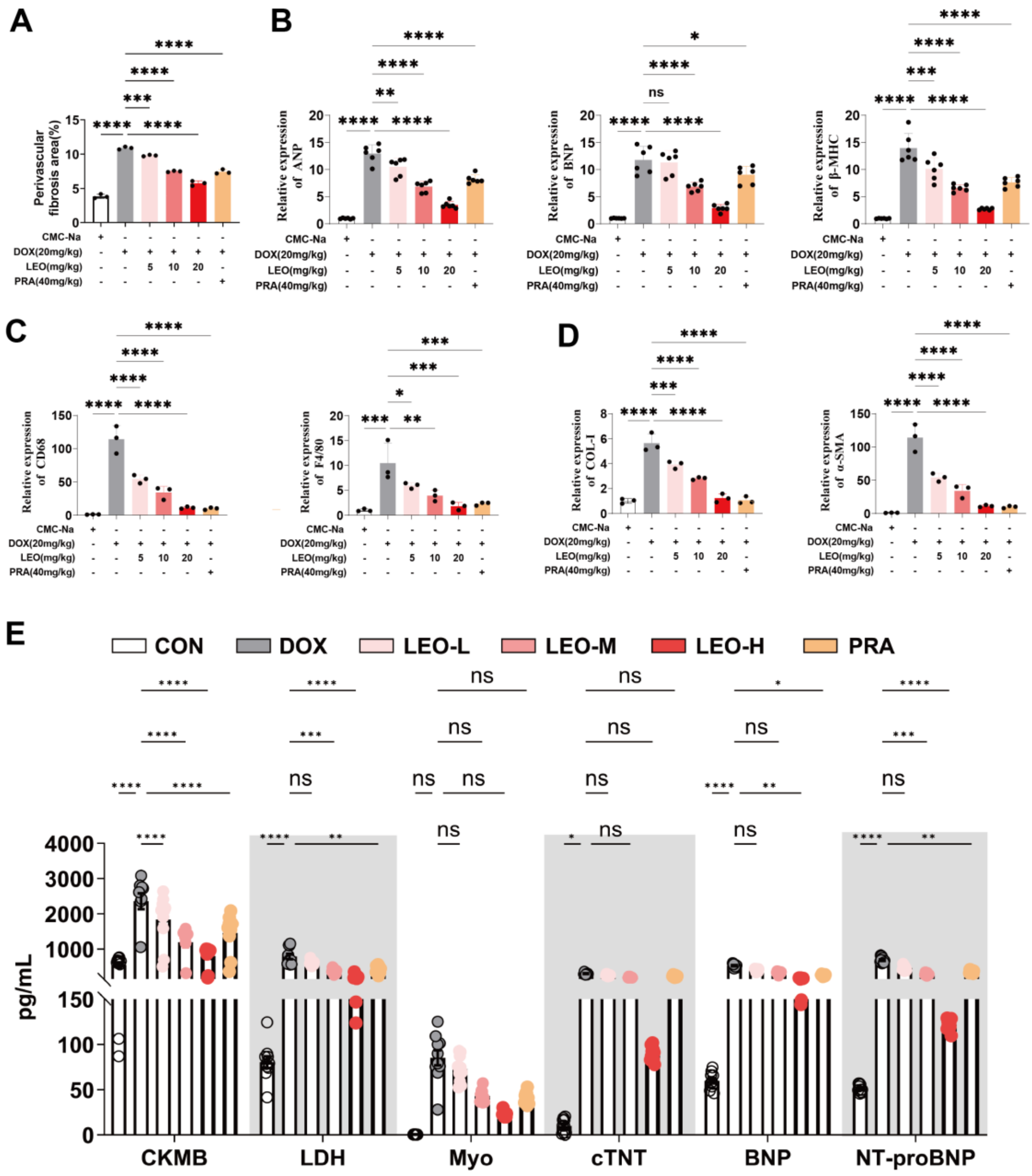

Figure S15. LEO improves cardiac inflammation, fibrosis, ventricular remodeling and serum cardiac enzymes in DIC mice. (A) Fibrotic deposition area in the hearts of DIC mice as shown by MASSON staining (n = 3). (B) mRNA expression levels of ANP, BNP, and β-MHC in the hearts of mice (n = 6). (C) Quantitative analysis of immunohistochemistry for CD68 and F4/80 in the hearts (n = 3). (D) Quantitative analysis of immunohistochemistry for α-SMA and COL-I in the hearts (n = 3). (E) Serum levels of CKMB, LDH, Myo, cTNT, and NT-proBNP (n = 10). $^{*}P < 0.05$, $^{**}P <$

0.01, $^{***}P < 0.001$, $^{****}P < 0.01$ and ns: Not significant.

17 LEO reduces the cGAS/STING signaling in the cardiac endothelium of DIC mice

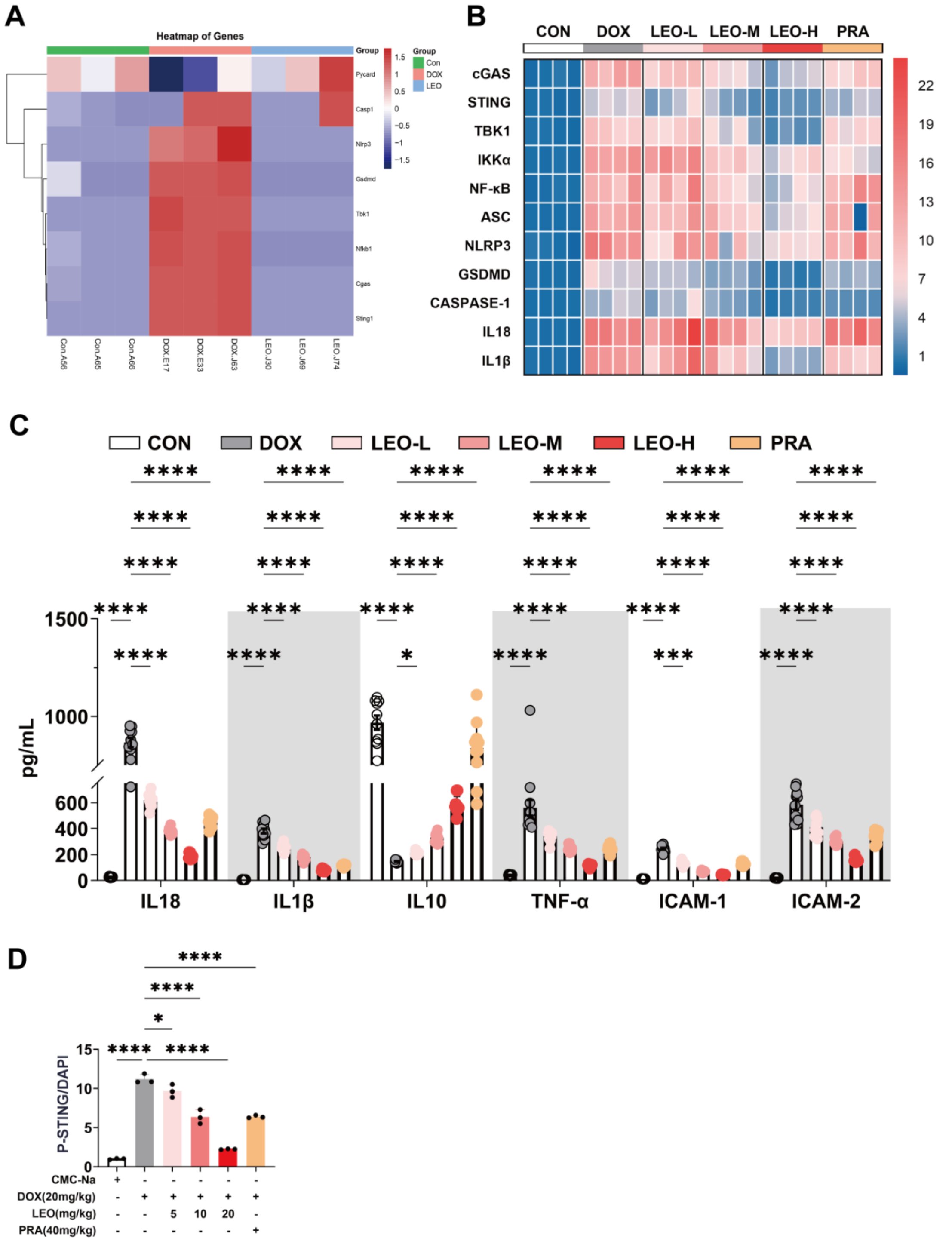

Figure S16. LEO reduces the cGAS/STING signaling in the cardiac endothelium of DIC mice. (A) Heatmap of differentially expressed genes among the CON group, DOX group, and LEO group (n = 3). (B) Relative mRNA expression levels of cGAS, STING, TBK1, IKKα, NF-κB, ASC, NLRP3, GSDMD, CASP1, IL18, and IL1β in the hearts of

mice (n = 6). (C) Serum levels of IL18, IL1β, TNFα, ICAM-1, ICAM-2, and IL10 (n = 10). (D) Immunofluorescence staining and quantitative analysis of P-STING and CD31 (n = 3). $^{*}P < 0.05$, $^{**}P < 0.01$, $^{***}P < 0.001$, $^{****}P < 0.01$ and ns: Not significant.

18 LEO enhances the functional capacity of CMVEC cells under DOX intervention

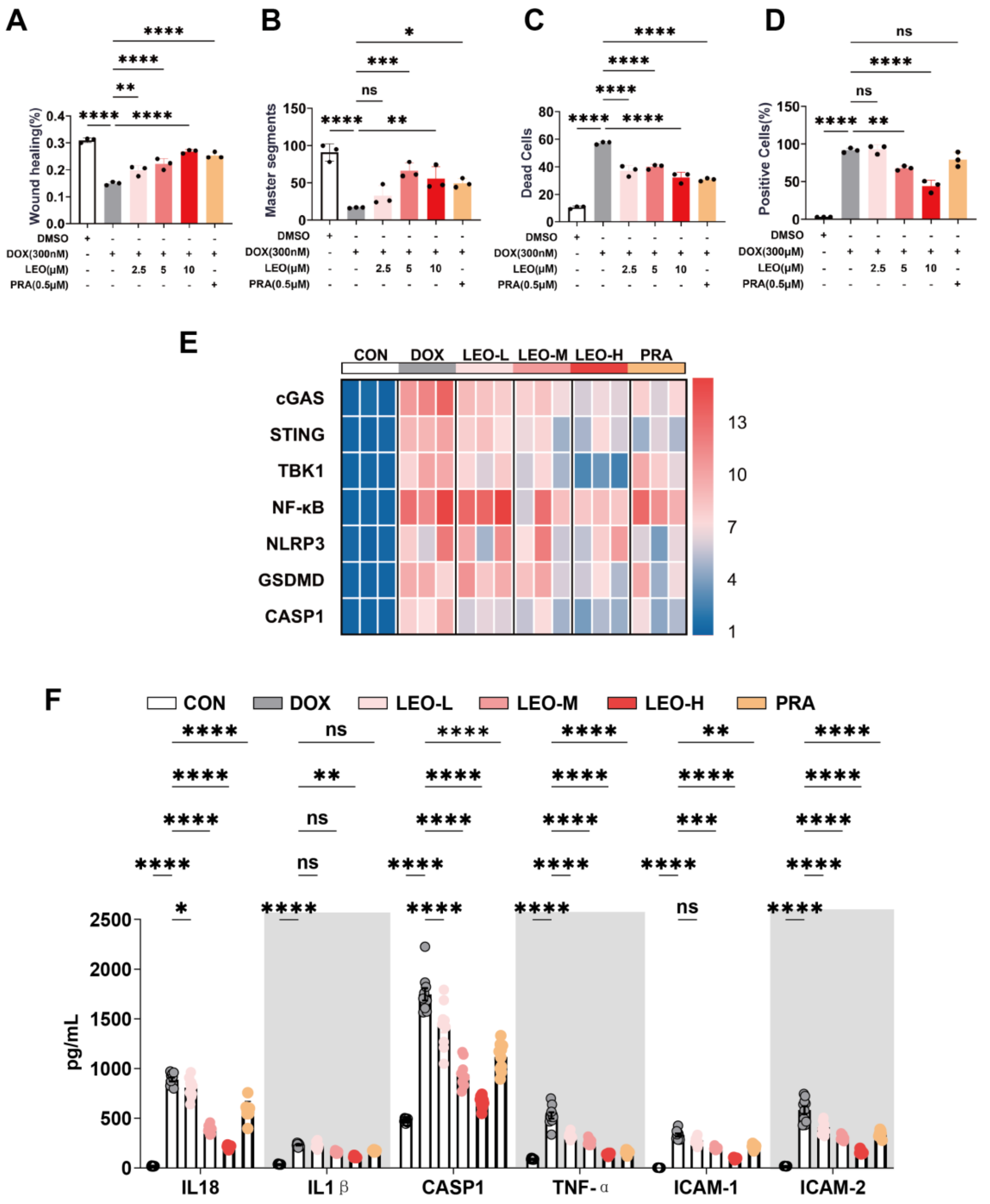

Figure S17. LEO enhances the functional capacity of CMVEC cells under DOX intervention. (A) Statistical analysis of the wound healing assay. (B) Statistical analysis of the tube formation assay. (C) Statistical analysis of Annexin V-FITC/PI flow cytometry. (D) TUNEL staining and statistical analysis. (E) Relative mRNA expression levels of cGAS, STING, TBK1, IKKα, NF-κB, ASC, NLRP3, GSDMD, CASP1, IL18, and IL1β. (F) Levels of IL18, IL1β, TNFα, ICAM-1, ICAM-2, and CASP1 in the

culture supernatant. $^{*}P < 0.05$, $^{**}P < 0.01$, $^{***}P < 0.001$, $^{****}P < 0.01$ and ns: Not significant.

19 Relative mRNA expression levels of CMVEC

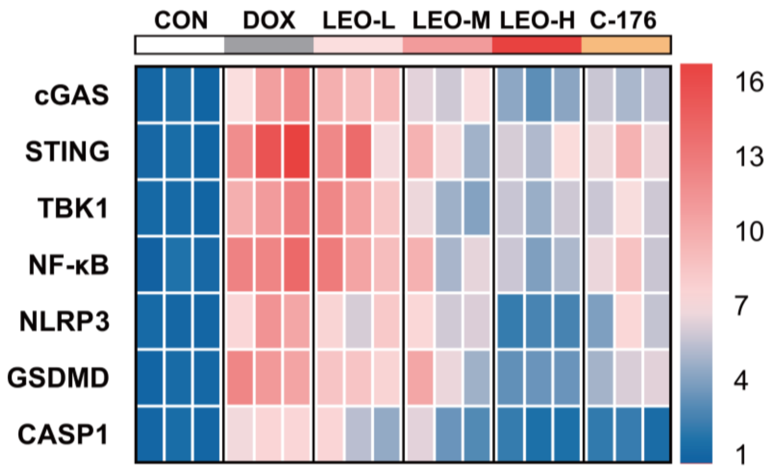

Figure S18. Relative mRNA expression levels of cGAS, STING, TBK1, IKKα, NF-κB, NLRP3, GSDMD, and CASP1 of CMVEC (n=3). $^{*}P < 0.05$, $^{**}P < 0.01$, $^{***}P < 0.001$, $^{****}P < 0.01$ and ns: Not significant.

20 The direct protective effect of LEO on H9C2

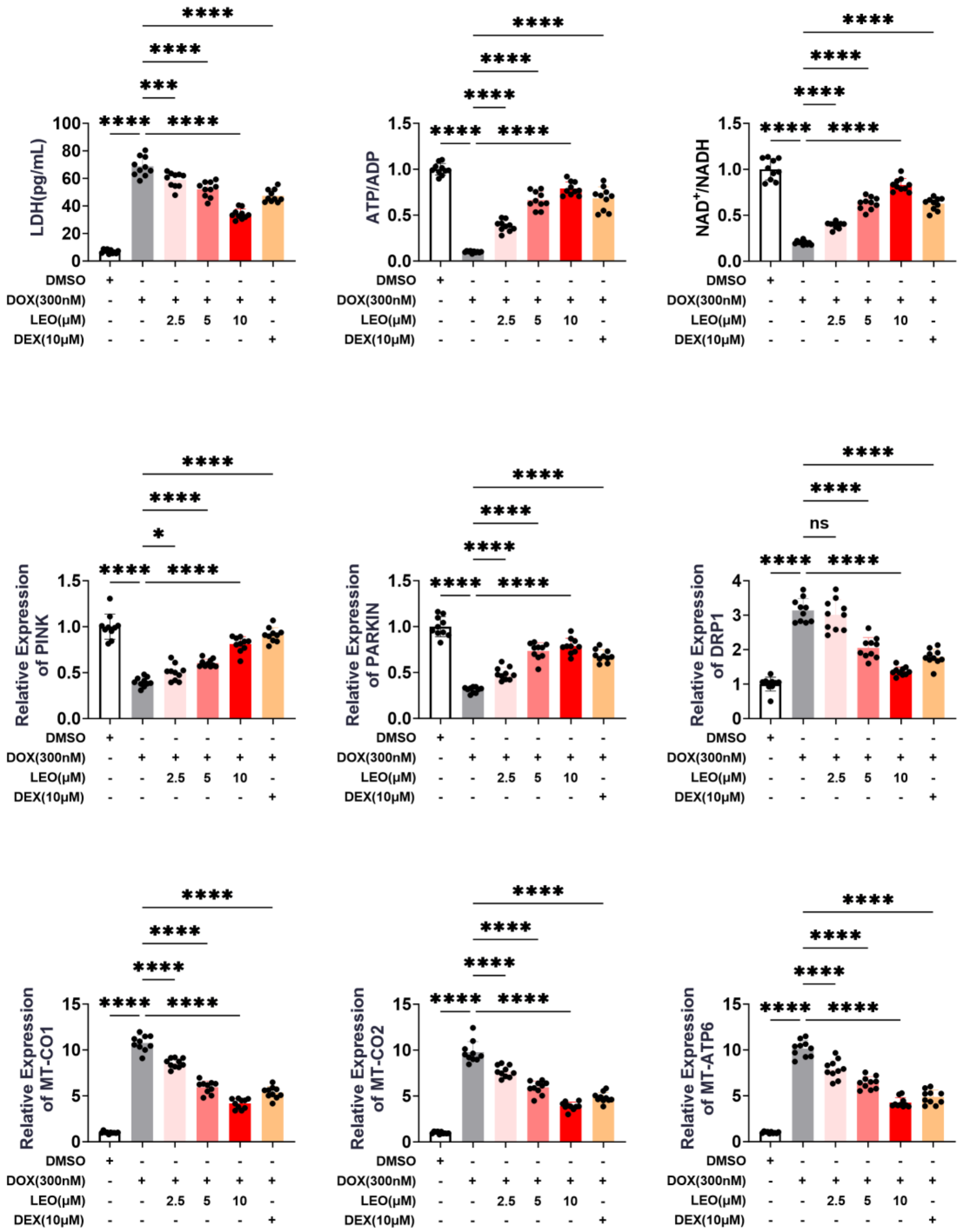

Figure S19. The direct protective effect of LEO on H9C2. DEX: Dexrazoxane. $^{*}P < 0.05$, $^{**}P < 0.01$, $^{***}P < 0.001$, $^{****}P < 0.01$ and ns: Not significant.

21 Genotyping of STING-KO mice

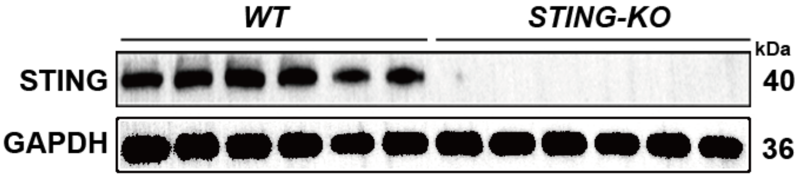

Figure S20. Genotyping of STING-KO mice.

22 dsDNA immunofluorescence in the hearts of DIC mice and H9C2

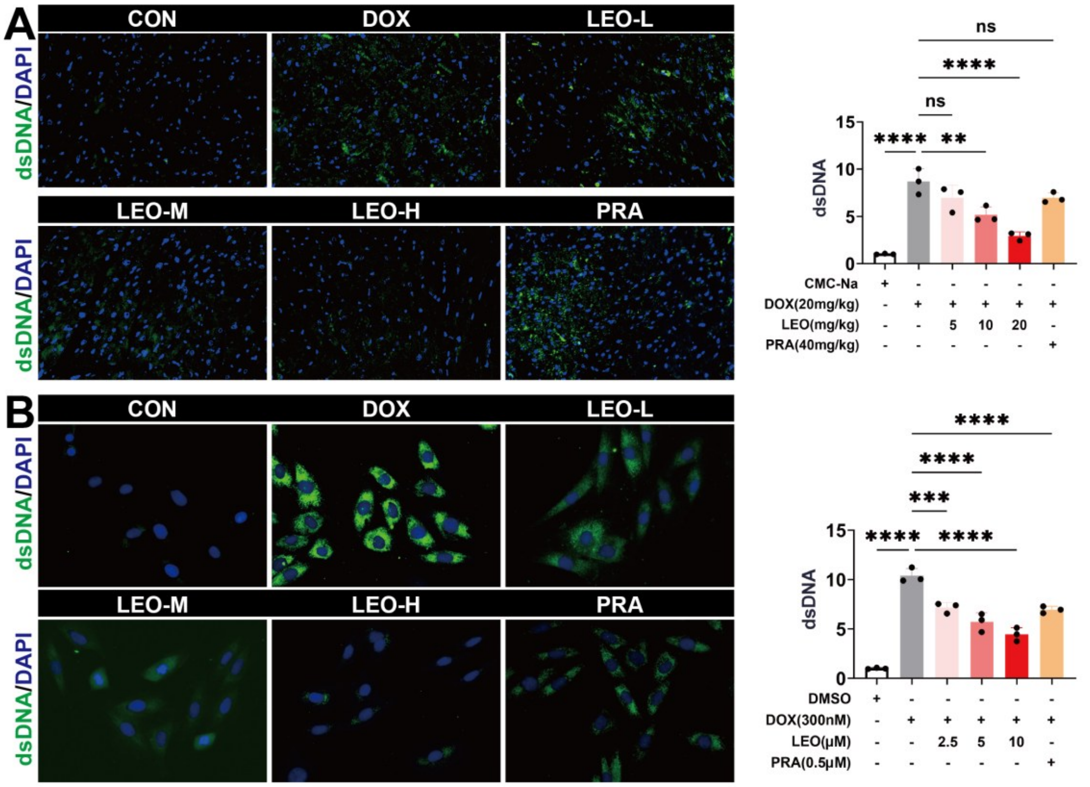

Figure S21. dsDNA immunofluorescence in the hearts of DIC mice and H9C2. (A) DIC mice. (B) H9C2.

23 Detailed parameters of the SPR experiment for LEO and STING proteins

Table S2. Detailed parameters of the SPR experiment for LEO and STING proteins

Parameters	Interpretation	Results
Ka(1/(M*s))	Binding rate constant	1.74E+02
kd (1/s)	Dissociation rate constant	1.25E-02
KD (M)	Affinity constant	7.18E-05
